# Experimental and Analytical Study on Concrete Mechanical Properties of Recycled Carbon Fibers from Wind Turbine Blades

**DOI:** 10.3390/ma18174105

**Published:** 2025-09-01

**Authors:** Julita Krassowska

**Affiliations:** Department of Building Structures, Bialystok University of Technology, 15-351 Bialystok, Poland; j.krassowska@pb.edu.pl

**Keywords:** recycled carbon fibers, fiber-reinforced concrete, mechanical properties, waste wind turbine blades

## Abstract

This study examines the effects of incorporating recycled carbon fibers obtained from decommissioned wind turbine blades into cementitious composites. An extensive experimental program was carried out, varying fiber content (0–8 kg/m^3^), fiber length (25, 38, 50 mm), water-to-cement ratio (0.4, 0.5), and cement type (CEM I 42.5, CEM II 42.5R/A-V). The mechanical properties of the fiber-reinforced concretes, including compressive strength, flexural strength, splitting tensile strength, and modulus of elasticity, were evaluated. The addition of recycled carbon fibers significantly improved flexural and splitting tensile strengths, with increases exceeding 60% and 100%, respectively, at the highest fiber dosage (8 kg/m^3^), attributed to efficient crack-bridging capability. Compressive strength was mainly influenced by the water-to-cement ratio, while the modulus of elasticity showed slight reductions in some mixes due to fiber clustering and increased micro-porosity. Regression analysis indicated that shorter fibers (25 mm) were more effective in enhancing flexural strength, whereas longer fibers (50 mm) improved splitting tensile strength. Classical predictive models generally underestimated the flexural capacity of recycled-carbon-fiber-reinforced concretes, highlighting the need for recalibration. Optical microscopy confirmed uniform fiber dispersion at lower dosages and a dominant pull-out failure mechanism. The findings demonstrate the feasibility of using recycled carbon fibers to enhance the mechanical performance of concrete while supporting sustainability through waste diversion and circular economy strategies.

## 1. Introduction

Concrete remains the most commonly used construction material in the world, primarily due to its high compressive strength, durability, and ease of shaping. However, its main disadvantage lies in its brittleness and low resistance to tension and bending, which promotes the initiation and propagation of cracks that may compromise the durability of structures. One way to improve these properties is through the use of fiber-reinforced concrete (FRC), in which fibers enhance the material’s ductility and inhibit the development of microcracks [1].

Until now, steel and polymer fibers have been the most commonly used in practice, but in recent years, increasing attention has been given to the potential use of recycled carbon fibers [2]. Decommissioned wind turbine blades and other composite waste represent a rich source of high-strength carbon fibers which, when incorporated into concrete, can not only improve its mechanical properties but also significantly reduce the amount of waste sent to landfills [3]. Thus, their application aligns with the principles of the circular economy and sustainable construction.

The addition of recycled carbon fiber waste to concrete has a significant effect on enhancing its tensile strength [4,5,6,7]. Studies show that introducing carbon fibers, even in small amounts, can improve the tensile behavior of concrete through crack bridging and enhanced interfacial bond strength between the fiber and the cement matrix. Research suggests that the optimal fiber content for increasing tensile strength typically ranges from 0.5% to 1.5% by volume. For instance, one study reported that a 1% volumetric content of recycled carbon fibers increased the splitting tensile strength by 14.3% compared to plain concrete [8]. Combining recycled carbon fibers with other additives such as silica fume can further boost tensile strength. For example, the addition of 0.6% recycled carbon fibers and 26.5 kg/m^3^ of silica fume led to an 80% increase in splitting tensile strength [9]. Carbon fibers recovered from Carbon-Fiber-Reinforced Polymer (CRFP) waste have also proven effective in enhancing tensile properties. In a study using microwave-assisted pyrolysis-recycled fibers, a 22.5% increase in tensile strength was observed at a fiber-to-cement mass ratio of 10% [10]. Uniform dispersion of recycled carbon fibers is crucial to maximizing flexural strength. One study demonstrated that uniform distribution of short carbon fibers, achieved using ultrasonic treatment and dispersing agents, led to a 19% increase in flexural strength [11]. Recycled carbon fibers have shown high effectiveness in improving flexural strength. For example, a study [12] using 1.5% recycled carbon fibers reported a 32.7% increase in flexural strength compared to fibreless concrete. The use of hybrid systems combining carbon fibers with other materials, such as aramid fibers, can further enhance flexural performance. One study found that a 50–50% blend of aramid and carbon fibers resulted in a significant improvement in flexural strength [13].

The compressive strength of concrete also improves with the incorporation of recycled carbon fiber waste. The optimal fiber content for enhancing compressive strength is typically around 1% by volume. One study reported a 20.9% increase in compressive strength at this fiber dosage [14]. Moreover, the fiber aspect ratio plays an important role—longer fibers provide better reinforcement effects [15]. Surface modification of recycled carbon fibers using nanoceramic coatings enhances their compatibility with the cement matrix, leading to increased compressive strength. For example, one study observed a 13% increase in compressive strength when using recycled carbon fibers treated with nanoclay [16]. The use of hybrid additives, such as silica fume in combination with recycled carbon fibers, also contributes to further enhancement of compressive strength. A study showed a 54.11% increase in compressive strength with the use of 0.6% recycled carbon fibers and 26.5 kg/m^3^ of silica fume [9].

Previous studies confirm that the incorporation of recycled carbon fibers into concrete can result in a significant increase in tensile and flexural strength due to effective crack bridging and improved energy absorption capacity. However, there is a considerable lack of data regarding the use of recycled carbon fibers recovered from composite waste, especially in the context of the combined influence of parameters such as fiber length, dosage, water-to-cement ratio, or cement type.

The aim of this study was therefore to comprehensively assess the influence of the content and length of recycled carbon fibers on the mechanical properties of concrete, including compressive strength, flexural strength, splitting tensile strength, and modulus of elasticity. The study also included a comparison of the behavior of fiber-reinforced concretes under different technological configurations (varied w/c ratios and cement types) in order to evaluate the interactions between these parameters. The experimental results were compared with existing empirical and standard-based models (ACI, RILEM, Ahmad & Shah) to assess their applicability to concretes reinforced with recycled carbon fibers. In addition, optical microscopy was used to analyze the distribution of fibers in the cement matrix, in order to better understand the mechanisms responsible for enhanced crack resistance. An analytical model was also developed to predict the flexural strength of concretes containing recycled carbon fibers.

The obtained results made it possible to evaluate the potential of recycled carbon fibers as a sustainable and effective reinforcement for next-generation concrete.

## 2. Materials and Methods

The research aimed to determine the impact of recycled carbon fibers recovered from advanced automotive and renewable energy sector products on the properties of cementitious concretes. The study was conducted in four stages:

**Stage 1—Cleaning of the polymer composite reinforced with recycled carbon fibers**

The tests used carbon fibers recovered from wind turbine blades made of polymer composite reinforced with recycled carbon fibers (CFRP—Carbon-Fiber-Reinforced Polymer). The thickness of the blade skin ranged from approximately 10 cm near the hub to 2–3 mm at the tips. In a 10-ton blade, the mass fraction of recycled carbon fibers was estimated at around 3 tons.

In the first stage, strips about 50 cm wide were cut from blade sections and mechanically pre-cleaned to remove adhesive residues and other composite components. The cleaned material was then cut into lengths of approximately 170 cm and prepared for pyrolysis at 500 °C for 60 min in an oxygen-free atmosphere. After pyrolysis, the fibers still contained a carbonized resin matrix, accounting for several percent of the recycled mass.

The next step was rolling, which reduced the strip width to around 10 mm without disturbing the parallel alignment of the fibers, facilitating further processing. The strips were then cut with guillotines, enabling automation and precise control of the target lengths. Finally, the fibers underwent an oxidation process in an oxygen-rich atmosphere at 500 °C for 30 min to completely remove residual carbonized resin, yielding fibers with near-complete purity.

**Stage 2—Development of the geometry of recycled carbon fibers and fiber production**

**Figure 1:**
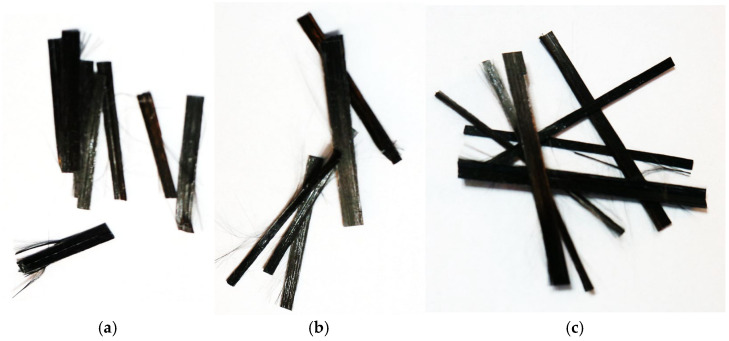
Recycled carbon fibers (recC) used in tests: (**a**) 25 mm, (**b**) 38 mm (**c**) 50 mm.

For the purposes of the study, the prepared strips with a width of approximately 10 mm were finally cut into segments with lengths of 25 mm, 38 mm, and 50 mm, resulting in a material suitable for use as macrofibre reinforcement in concretes modified with recycled carbon fibers (Figure 1).

**Stage 3—Testing the mechanical properties of recycled carbon macrofibers**

The third stage involved analysis of the chemical composition, microstructure, and mechanical properties of the recycled carbon fibers.

Initially, the geometrical characteristics of the macrofibers were verified in accordance with [17], with the aim of determining the average values of surface area, diameter, and perimeter. Uniaxial tensile testing of the examined fibers was then carried out to determine the average tensile strength, the mean longitudinal modulus of elasticity, and the ultimate strain of the macrofibers in accordance with [17].

**Stage 4—Determination of the strength parameters of concretes with recycled carbon fibers**

The research program included the development of concrete mix compositions and the assessment of both fresh mix properties and mechanical characteristics of hardened concrete, such as compressive strength, flexural strength, splitting tensile strength, and Modulus of elasticity. These tests were conducted to compare the behavior of fiber-reinforced concretes with reference mixtures.

**Figure 2:**
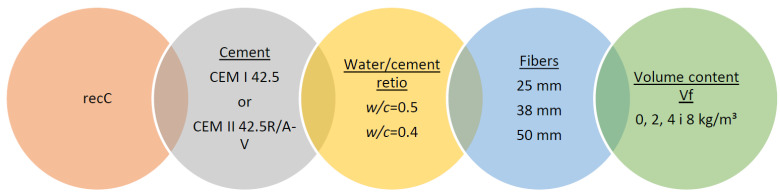
Designation of Test Series.

Three main groups of concrete mixes were produced, each reinforced with randomly fiber recycled carbon fiber strips (minibars) of lengths 25 mm, 38 mm, and 50 mm. Two types of cement were used: Cemex CEM I 42.5 and CEM II 42.5R/A-V (Cemex Poland, Warsaw, Poland). Mixes were prepared with two different water-to-cement ratios (*w*/*c* = 0.50 and 0.40) and four fiber volume contents: 0, 2, 4, and 8 kg/m^3^. The designation of the test series is shown in Figure 2.

This experimental design enabled a detailed analysis of the effects of fiber length and dosage, cement type, and *w*/*c* ratio on the mechanical properties of the tested concretes. In total, 48 series of concrete mixes were prepared, allowing for a comprehensive evaluation of the performance of recycled carbon fiber reinforcement under various technological conditions.

### 2.1. Materials

Two base mix designs of cement concretes were used in the study, differing in water-to-cement ratio (*w*/*c*), which was set at 0.5 and 0.4, respectively, and in the type of cement: CEM I 42.5R or CEM II 42.5R/A-V (320 kg/m^3^). The mix proportions are provided in Table 1. In both cases, the mixtures were modified with recycled carbon fibers in amounts corresponding to volumetric contents *V_f_* = 0, 2, 4, and 8 kg/m^3^, partially replacing the aggregate accordingly. A high-range water-reducing admixture (Sika Sikacem Superplast, Sika Poland, Warsaw, Poland) was used, dosed at 1–2% of the cement mass. This superplasticizer allowed for a significant reduction in water content while maintaining adequate workability.

### 2.2. Testing Procedure

#### 2.2.1. Chemical Composition Analysis of Recycled Carbon Fibers

The analysis was performed using an FTIR spectrometer equipped with an ATR accessory featuring a diamond crystal. Measurements were conducted in the spectral range of 4000–600 cm^−1^ at an incident angle of 45°. Since the study employed the ATR accessory, the samples were analyzed in the form of entire fiber strips without the need for additional sample preparation, which significantly contributed to the quality of the obtained spectra.

#### 2.2.2. Mechanical Properties Testing of Recycled Carbon Fibers

**Figure 3:**
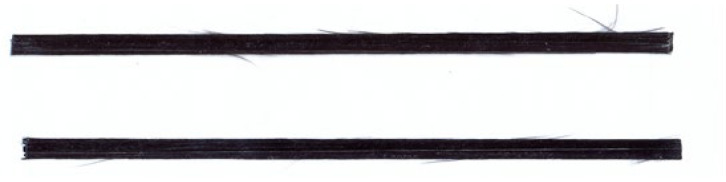
Recycled carbon fiber strips subjected to tensile testing.

The characterization of the macrofibers began with geometric measurements, including fiber length, diameter, and perimeter, to determine the average cross-sectional area in accordance with the guidelines provided in [17]. Subsequently, uniaxial tensile tests were conducted to determine the key mechanical parameters: average tensile strength, average longitudinal modulus of elasticity, and ultimate strain of the macrofibers. These tests followed the procedures described in [18]. Tensile resistance tests were performed using an testing machine at a strain rate of 0.5 mm/min. The specimens consisted of recycled carbon fiber strips with a length of 195 mm, width of 6 mm, and thickness of 0.1 mm (Figure 3). The samples were subjected to uniaxial tensile loading along the fiber direction. Elongation was recorded based on the displacement rate of the machine’s upper crosshead (0.5 mm/min), until a drop in force was observed.

#### 2.2.3. Slump Test of Fresh Concrete Mix

The consistency of the concrete mix was evaluated using the slump test (Abrams cone), in accordance with the requirements of EN 12350 [19]. The test involved filling a cone with a height of 300 mm, a base diameter of 200 mm, and a top diameter of 100 mm with concrete mix in three equal layers, each compacted by 25 strokes with a steel rod of 16 mm diameter. After leveling the surface, the cone was lifted vertically and smoothly within 5–10 s, allowing the mix to slump freely. The consistency was determined by measuring the height difference between the original height of the cone and the highest point of the slumped mix, with an accuracy of 5 mm. The measured slump value indicates the workability of the mix and allows for comparison of its rheological properties depending on the composition and parameters of the applied fibers.

#### 2.2.4. Density Testing of Concrete

The apparent density test was conducted to determine the effect of fiber content, cement type, and water-to-cement (*w*/*c*) ratio on the density of hardened concrete. The measurement was carried out in accordance with EN 12390-7 [20]. Cube specimens with dimensions of 150 mm × 150 mm × 150 mm were weighed using an electronic balance with an accuracy of 1 g, and their volume was determined geometrically.

#### 2.2.5. Compressive Strength Testing of Concrete

Compressive strength tests were performed in accordance with EN 12390-3 [21], using cube specimens with 100 mm sides. Failure load measurements were carried out using a testing machine with a maximum capacity of 3000 kN. The loading rate was 0.5 MPa/s.

The compressive strength $f_{c}$ was calculated using the following formula:

(1) $$f_{c}=\frac{P}{A_{c}},$$

where *P*—maximum load at failure; *A_c_*—cross-sectional area of the sample

#### 2.2.6. Flexural Tensile Strength Testing of Concrete

The flexural tensile strength was determined on specimens with dimensions of 100 mm× 100 mm× 400 mm, in accordance with EN 12390-5 [22]. The beams were loaded with a concentrated force *P* applied at mid-span. The failure load was measured using a testing machine with a maximum capacity of 100 kN. The loading rate was 0.05 MPa/s.

The flexural tensile strength $f_{ct}$ was calculated using the following formula:

(2) $$f_{ct}=\frac{3Pl}{2d_{1}d_{2}^{2}},$$

where *P*—maximum load; *l*—spacing of supporting rollers; *d*_1/2_—dimensions of the cross-section of the sample.

#### 2.2.7. Splitting Tensile Strength Testing of Concrete

**Figure 4:**
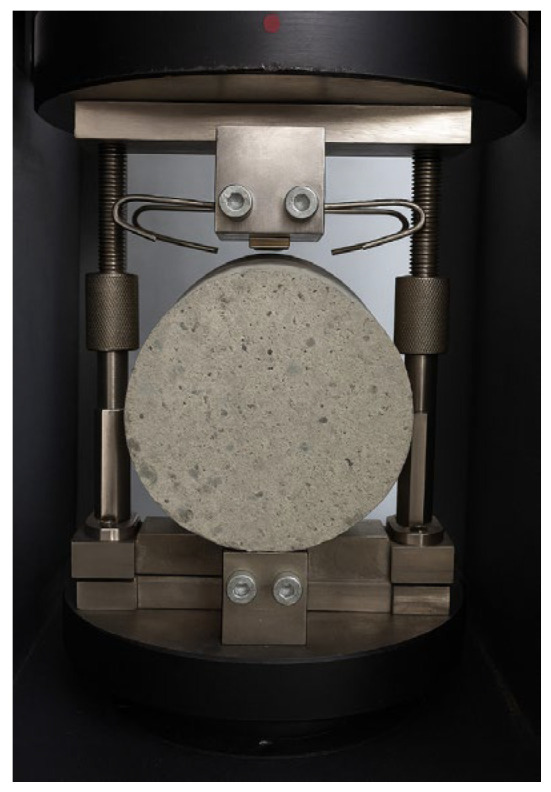
Sample prepared for splitting tensile strength testing according to EN 12390-6 [23].

The splitting tensile strength was tested in accordance with EN 12390-6 [23]. The tests were performed using cylindrical specimens with a height h = 300 mm and diameter d = 150 mm. A steel frame was used for proper positioning of the specimens in the press (Figure 4). The failure load was recorded using a testing machine with a maximum capacity of 3000 kN. The loading rate was 0.05 MPa/s. Each test series consisted of three cylindrical specimens.

The splitting tensile strength of the specimens *f_cf_* was calculated using the following formula:

Splitting Tensile Strength was calculated using the following formula:

(3) $$f_{cf}=\frac{2P}{\pi Ld}\;,$$

where P—maximum applied load, *l*—length of the contact line between the loading strips and the specimen, *d*—cross-sectional dimension of the specimen.

#### 2.2.8. Testing of Concrete Modulus of Elasticity

The longitudinal modulus of elasticity was determined in accordance with EN 12390-13 [24], using cylindrical specimens with a diameter of 150 mm and a height of 300 mm. The tests were performed on a CONTROLS C86Z00 testing machine with a maximum load capacity of 3000 kN and mechanical extensometers.

Method B of specimen loading, as described in EN 12390-13 [24], was used in the testing procedure.

The longitudinal modulus of elasticity was calculated using the formula:

(4) $$E_{c,s}=\frac{∆\sigma }{∆\varepsilon_{s}}=\frac{\sigma_{a}^{m}-\sigma_{p}^{m}}{\varepsilon_{a,3}-\varepsilon_{p,2}}\;,$$

where ∆σ—difference between measured stresses, $∆\varepsilon_{s}$—difference in strains during the third loading cycle, $\sigma_{a}^{m}$—stress corresponding to the nominal upper stress, $\sigma_{p}^{m}$—measured stress corresponding to the nominal preload stress, $\varepsilon_{a,3},\varepsilon_{p,2}$—mean strains under preload and final load in the *n* loading cycle.

## 3. Results

### 3.1. Analysis of the Composition and Mechanical Parameters of Carbon Fibers

**Figure 5:**
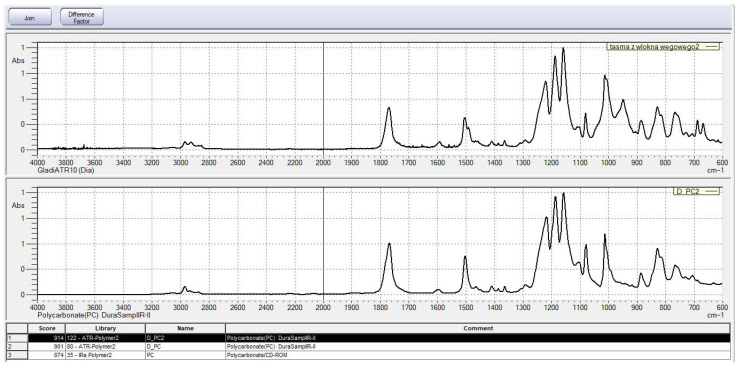
Spectra of the tested sample and the standard obtained from FTIR spectroscopic studies.

Using the spectroscopic reference spectrum database of the measurement device, the obtained results were identified, indicating the presence of polycarbonate (PC) on the surface of the recycled carbon fibers. This compound acts as a binder connecting individual fibers within the strips. An image of the spectrum obtained for the tested samples, along with the reference spectrum, is presented in Figure 5.

Figure 5 presents a comparison between the spectrum of the sample in the form of a carbon fiber strip (upper curve) and the reference spectrum of polycarbonate (lower curve), sourced from the database. The spectra exhibit a high degree of correspondence in terms of characteristic absorption bands, confirming the presence of polycarbonate as the main component of the organic phase in the tested sample.

Intense bands were observed in the following regions:

- 1770–1780 cm^−1^, corresponding to the stretching vibrations of carbonyl groups (C=O),
- 1500–1600 cm^−1^, associated with aromatic ring vibrations,
- 1200–1300 cm^−1^, typical for the stretching vibrations of C–O bonds in the ester structure of polycarbonate.

Additionally, bands characteristic of skeletal vibrations of aromatic systems are visible below 1000 cm^−1^. The comparative analysis with the spectral library indicated the highest match with the structure of polycarbonate (PC), achieving fit scores in the range of 874–914, which clearly confirms the identification of the matrix material. It can therefore be concluded that the matrix of the composite from which the recycled carbon fibers were obtained was polycarbonate.

**Figure 6:**
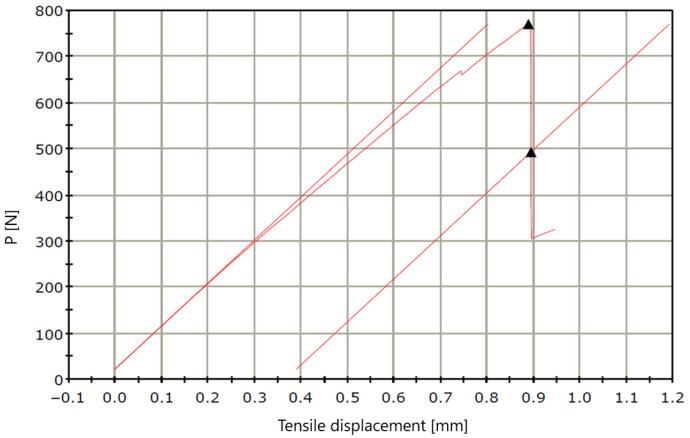
Tensile test measurement curve for carbon fiber strips.

An image of the tensile test curve of recycled carbon fiber strips is shown in Figure 6.

**Figure 7:**
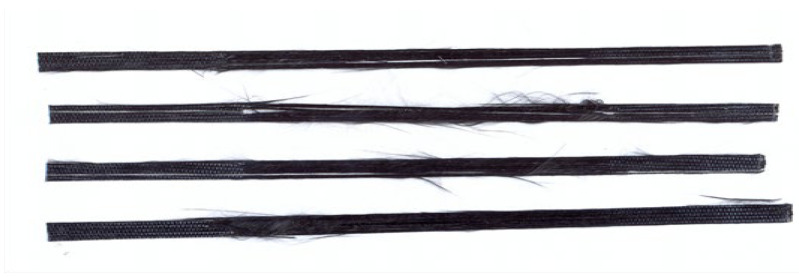
Recycled carbon fiber strips after tensile testing.

After the tensile test was performed, the specimens did not experience complete rupture but rather fraying of individual fibers only (Figure 7).

The measurement results obtained are presented in Table 2.

The average tensile strength *R_m_* was 1168.5 MPa, with individual specimen values ranging from 1093.5 MPa to 1283.5 MPa. The average Modulus of elasticity, determined from the initial linear portion of the force–displacement curve, was 317 GPa, indicating high stiffness of the carbon fibers. The average relative elongation at tensile strength was 0.395%, while the average true stress at maximum displacement during tension reached 450.4 MPa.

It is worth noting the relatively low strain values at *R_m_*, which is typical for brittle materials with a high modulus of elasticity and confirms the characteristic tensile behavior of recycled carbon fibers. The obtained results indicate that the recovered carbon fibers retained very good mechanical and elastic properties, enabling their effective use as fiber reinforcement in cement-based composites.

### 3.2. Analysis of Concrete Mix and Hardened Concrete Properties

#### 3.2.1. Effect of Fibers on Concrete Mix Consistency

**Figure 8:**
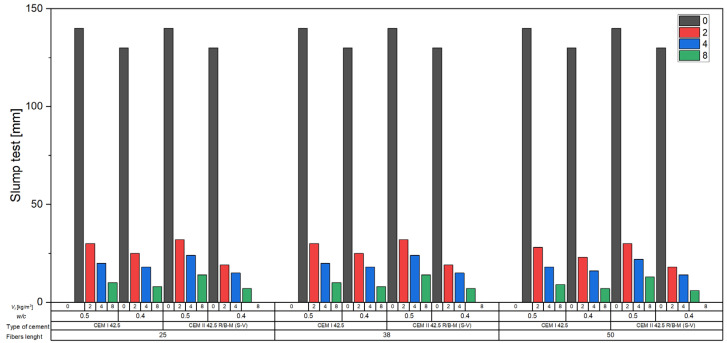
Workability of concrete mixes.

Figure 8 presents the results of the slump test for a series of concrete mixes containing recycled carbon fibers with lengths of 25, 38, and 50 mm, introduced in various volume fractions (*V_f_* = 0–8%). The mixes were prepared using two types of cement (CEM I 42.5 and CEM II 42.5R/A-V) and two water-to-cement ratios (*w*/*c* = 0.5 and 0.4).

Based on the data obtained, a clear effect of fiber content on the workability of the concrete mix was observed. For all tested variants, there was a systematic decrease in slump with increasing fiber content from 0 kg/m^3^ to 8 kg/m^3^ by volume. For example, in the case of mixes with CEM I cement and a w/c ratio of 0.5, the slump decreased from 140 mm (S3) in the fiber-free mix to 30 mm (S1) for *V_f_* = 2 kg/m^3^, 20 mm (S1) for *V_f_* = 4 kg/m^3^, and 10 mm (S1) for *V_f_* = 8 kg/m^3^ in the series with 25 mm fibers. Similar trends were observed for fibers with lengths of 38 and 50 mm; however, mixes with the longest fibers (50 mm) showed even lower slump values, reaching only 6–9 mm, indicating a significant reduction in workability.

The influence of fiber length was also evident in mixes with a lower w/c ratio of 0.4. Despite the reduced water content, fiber-free mixes still exhibited high slump values (130 mm, class S3). However, the addition of 2 kg/m^3^ of fibers led to a slump decrease to 18–25 mm (S1), and for 8 kg/m^3^, it dropped to just 6–8 mm, regardless of fiber length.

When comparing mixes with CEM I and CEM II cement, no significant differences in slump were observed for analogous compositions, suggesting that the type of cement had less impact on workability than fiber content and length.

The increase in recycled carbon fiber content in the concrete mix significantly reduced its consistency, causing a transition from class S3 (100–150 mm) for fiber-free mixes to class S1 (10–40 mm) for mixes with the highest fiber dosage. This is a result of increased internal friction and the tendency of fibers to entangle and retain water, which is further intensified with fiber length.

#### 3.2.2. Effect of Fibers on Density

**Figure 9:**
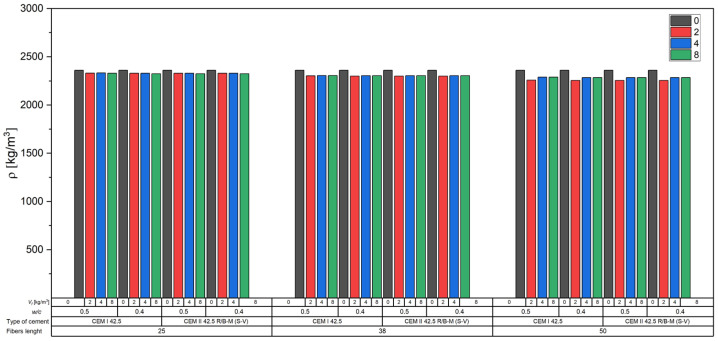
Relationship between density and concrete series.

Figure 9 presents the results of density measurements (kg/m^3^) of hardened concrete for all test series, which varied in terms of cement type (CEM I 42.5 and CEM II 42.5 R/B-M (S-V)), water-to-cement ratio (*w*/*c* = 0.4 and 0.5), and fiber content *V_f_* (0, 2, 4, and 8 kg/m^3^).

The density of all concrete mixes ranged from approximately 2255 kg/m^3^ to 2360 kg/m^3^. The highest values (2360 kg/m^3^) were consistently recorded for reference mixes without any fiber addition (*V_f_* = 0 kg/m^3^).

The incorporation of fibers caused a gradual decrease in concrete density—the lowest values were obtained for the highest fiber dosage (8 kg/m^3^), reaching about 2285 kg/m^3^ in the CEM II series with *w*/*c* = 0.4.

The influence of the w/c ratio was relatively minor. However, slightly lower densities were observed in the *w*/*c* = 0.4 mixes compared to *w*/*c* = 0.5 for the same fiber content.

Differences between concretes made with CEM I and CEM II R/B-M (S-V) cement were minimal and fell within the margin of measurement error, indicating similar mix compactness and matrix tightness regardless of the cement type.

Fibers typically have a lower density than the cementitious matrix and aggregate. Carbon fibers have a density of approximately 1950 kg/m^3^, which is considerably lower than that of aggregate (2700 kg/m^3^) and the hardened cement matrix (approximately 2300–2400 kg/m^3^). As fibers replace heavier mix components (mainly mortar and fine aggregate), their inclusion leads to a reduction in overall density.

Furthermore, the presence of fibers increases the risk of microvoid formation in the concrete. Especially at higher fiber dosages (4 and 8 kg/m^3^), they may hinder proper compaction of the mix, leading to a slightly higher air content and, consequently, reduced density.

#### 3.2.3. Effect of Fibers on Concrete Compressive Strength

**Figure 10:**
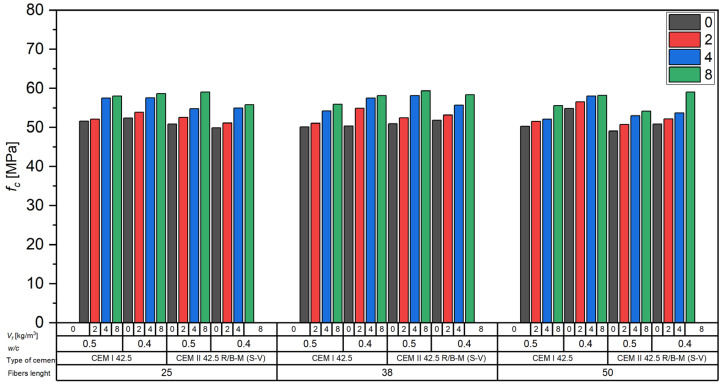
Influence of carbon fibers on the compressive strength of concrete.

The influence of carbon fiber reinforcement on the compressive strength of concrete was analyzed using cube specimens, and the results are presented in Figure 10.

The compressive strength (*f_c_*) of the tested concrete mixes varied depending on the type of cement, the water-to-cement ratio (*w*/*c*), fiber length, and fiber content (*V_f_*). Compressive strength values did not show a consistent trend with respect to the water-to-cement ratio. In some cases, mixtures with w/c = 0.50 exhibited comparable or even higher fc values than those with *w*/*c* = 0.40, particularly for CEM II 42.5 R/B-M (S-V) and 25 mm fibers.

Based on the data shown in Figure 10, the following observations can be made:

- For concretes made with CEM I 42.5, compressive strength ranged approximately from 51 to 59 MPa, with higher values (ca. 58–59 MPa) recorded for mixes with *w*/*c* = 0.4;
- A similar trend was observed in concretes made with CEM II 42.5 R/B-M (S-V), where again, higher *f_c_* values (exceeding 58 MPa) were observed for *w*/*c* = 0.4;
- Fiber length (25, 38, 50 mm) did not result in any significant differences in the compressive strength trends.

The addition of recycled carbon fibers in the range of 2 to 8 kg/m^3^ consistently resulted in higher compressive strength (*f_c_*) compared to the unreinforced concrete. At the highest dosage (8 kg/m^3^), the increase in fc was clearly visible in all tested series. These results indicate that, although the *w*/*c* ratio remained the dominant factor influencing compressive strength, the presence of recycled fibers also contributed positively to strength development across all dosages.

#### 3.2.4. Effect of Fibers on the Flexural Tensile Strength of Concrete

**Figure 11:**
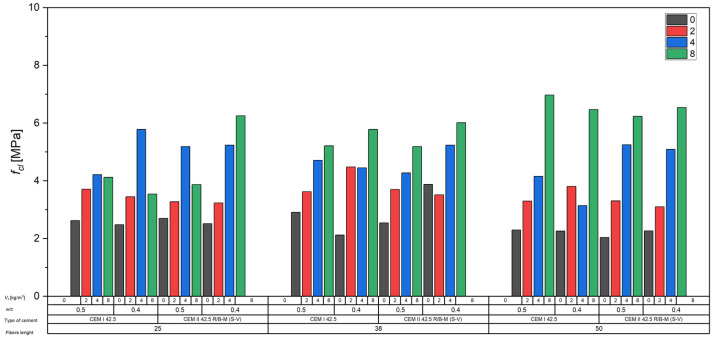
Influence of carbon fibers on the flexural tensile strength of concrete.

The analysis of the test results clearly demonstrated the significant influence of fiber content on the flexural tensile strength of concrete (*f_ct_*). In most cases, the addition of fibers led to a substantial increase in flexural strength compared to reference concretes without fiber reinforcement (Figure 11).

For concretes made with CEM I 42.5 cement and a water-to-cement ratio of *w*/*c* = 0.5, the flexural strength *f_ct_* increased from approximately 2.6 MPa for the unreinforced concrete to over 4 MPa at a fiber content of 8 kg/m^3^, and in some series, even up to 6.9 MPa. Similar trends were observed for concretes made with CEM II 42.5 R/B-M (S-V), where the flexural strength rose from 2.0 to 2.7 MPa for unreinforced concrete to values exceeding 6.0 MPa at the highest fiber dosage.

A reinforcing effect of fiber reinforcement was also observed in concretes with a reduced water-to-cement ratio of *w*/*c* = 0.4, where fibers contributed to improved *f_ct_* values compared to the respective reference mixes. The most favorable results were achieved in the 4 to 8 kg/m^3^ fiber dosage range, where the greatest increases in flexural strength were recorded.

**Figure 12:**
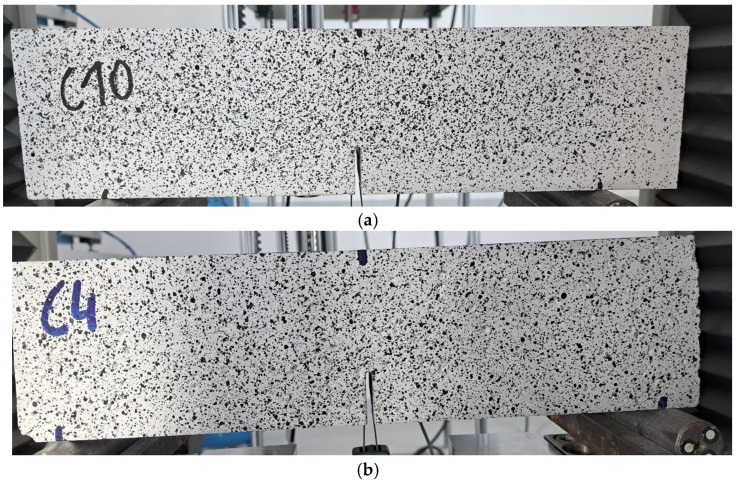

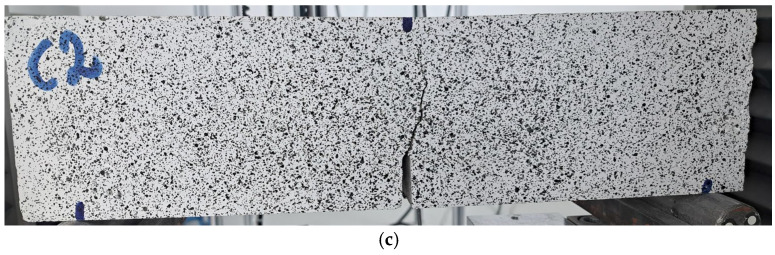
Typical failure modes of concrete beams reinforced with recycled carbon fibers: in series: (**a**) recC—CEM II 42.5R/A-V—0.5-2-50 (**b**) recC—CEM I 42.5R—0.5-8-35 (**c**) recC—CEM I 42.5R—0.5-4-25.

Figure 12 presents typical crack patterns observed in fiber-reinforced concrete beams containing recycled carbon fibers after four-point bending tests. All beams exhibited typical failure modes for fiber-reinforced concretes: instead of a sudden brittle failure, a gradual development of a single dominant crack was observed. This behavior confirms the favorable crack-bridging action of carbon fibers and the increased ductility of the composite.

#### 3.2.5. Effect of Fibers on the Splitting Tensile Strength of Concrete

**Figure 13:**
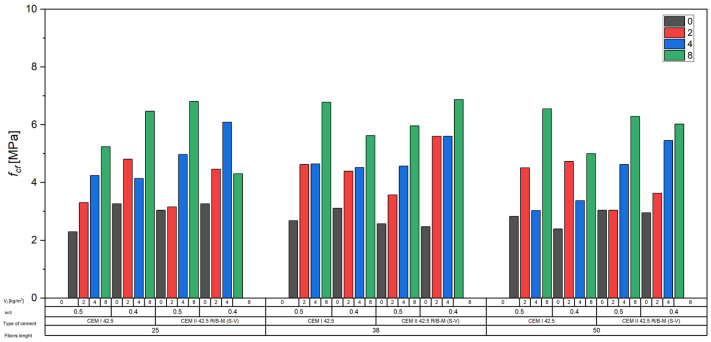
Influence of carbon fibers on the splitting tensile strength of concrete.

The results clearly demonstrate that the use of recycled carbon fibers led to a significant increase in the splitting tensile strength *f_cf_* of concrete compared to the reference fiber-free mixtures (Figure 13).

Concretes made with CEM I 42.5 cement and a w/c ratio of 0.5 showed an increase in *f_cf_* from approximately 2.3–2.8 MPa for the unreinforced mixes to values exceeding 6 MPa at the highest fiber content (8 kg/m^3^). The average increases reached 150–200% relative to the reference values. In concretes with *w*/*c* = 0.4, the effect was even more pronounced—for instance, mixes with 8 kg/m^3^ of fibers reached splitting tensile strengths of around 6.5 MPa, whereas the corresponding reference concrete ranged between 2.4 and 3.3 MPa.

Concretes with CEM II 42.5 R/B-M (S-V) also showed a distinct effect of fiber inclusion. For *w*/*c* = 0.5, the *f_cf_* increased from approximately 2.6–3.1 MPa (0 kg/m^3^) to as much as 6.8 MPa (8 kg/m^3^). In the *w*/*c* = 0.4 series, final values reached around 6.9 MPa, representing more than a twofold increase compared to the unreinforced concrete.

#### 3.2.6. Effect of Fibers on Modulus of Elasticity

**Figure 14:**
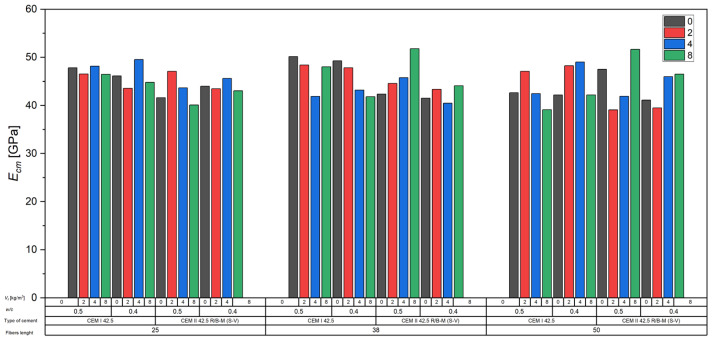
Influence of carbon fibers on Modulus of elasticity of concrete.

The analysis of the obtained results for the modulus of elasticity *E_cm_* of concrete showed that the incorporation of recycled carbon fibers within the studied volumetric ranges did not have a clear effect on the average Modulus of elasticity compared to the reference concretes without fibers (Figure 14). The addition of fibers caused slight increases or decreases in *E_cm_* values in some series, but these changes generally remained within the standard deviation limits for the respective groups.

At the same time, it was observed that fiber addition often led to increased variability in the results, as reflected in higher standard deviations, particularly at higher fiber contents. This may be attributed to difficulties in achieving uniform fiber dispersion within the cement matrix and local structural inhomogeneities in the concrete, which can significantly influence its stiffness.

The results suggest that while carbon fibers in the investigated system do not substantially alter the macro-scale Modulus of elasticity of concrete, they may contribute to local elastic heterogeneity, which is relevant in the context of crack initiation and propagation analysis.

From a mechanical perspective, these local stiffness variations are relevant because they can influence crack initiation and propagation mechanisms. Non-uniform stiffness distribution in the microstructure may lead to localized stress concentrations, affecting the material’s post-cracking behavior and residual load-bearing capacity. While recycled carbon fibers in the investigated system do not significantly alter the overall modulus of elasticity, their potential impact on micro-scale mechanical performance should be considered in future analyses.

### 3.3. Miscoscopy Analysis

**Figure 15:**
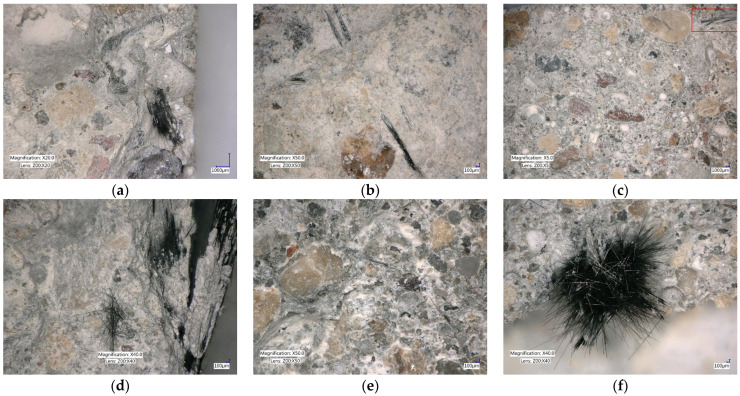
Photographs of the fractures of the specimens: (**a**) recC2-50—CEM I 42.5-0.5-2 (**b**) recC11-38—CEM II 42.5R/A-V-0.5-4 (**c**) recC4-50—CEM I 42.5-0.5-8 (The red box in the figure indicates the location of the fiber in the concrete.) (**d**) recC16-25—CEM II 42.5R/A-V—0.4-8 (**e**) recC10-25-EM II 42.5R/A-V-0.5-2 (**f**) recC11-38—CEM I 42.5-0.5-4.

Fracture images of the specimens reinforced with recycled carbon fibers are presented in Figure 15. In the case of specimens with chopped basalt fibers, fibers were only clearly visible at a dosage of 8 kg/m^3^; at lower dosages, the fibers could not be observed. In contrast, the use of basalt minibars allowed fiber visibility even at the lower content of 2 kg/m^3^.

Microscopic images taken using the Keyence optical microscope at various magnifications (×5, ×10, and ×50) enabled a comprehensive assessment of the distribution of recycled carbon fibers in the concrete matrix and the damage mechanisms within the fracture zone.

It was found that in most cases, the fibers were relatively uniformly distributed throughout the concrete volume, without clearly defined areas lacking fiber reinforcement. At a fiber dosage of 2 kg/m^3^, the carbon fibers appeared well-fiber as thin, single filaments visible both in the cement matrix and near the coarse aggregate particles. This fiber distribution promotes uniform stress transfer in the concrete and reduces localized reinforcement concentration.

In contrast, in specimens with higher fiber contents (4 and 8 kg/m^3^), a tendency for fibers to form wider bands and local bundles was observed. These bundles often filled the cement paste shell around several fibers. This arrangement indicates a natural tendency for partial agglomeration at higher dosages, which, however, did not lead to clear structural defects or compromise the homogeneity of the concrete within the studied range.

Numerous fibers protruding from the cement matrix were observed on the fracture surfaces, confirming the dominance of the partial pull-out mechanism (as seen in recC16-25-CEM II 42.5R/A-V-0.4-8), where fibers gradually disengage from the cement matrix without complete breakage. This damage mechanism is favorable, as it effectively dissipates fracture energy and slows the propagation of cracks under tensile loads.

In addition to the pull-out phenomenon, other failure mechanisms of fibers in cement composites were also observed, such as fiber rupture (recC11-38-CEM I 42.5-0.5-4), debonding of the cement matrix from the fiber (recC11-38-CEM II 42.5R/A-V-0.5-4), microindentations and crushing of the cement paste around the fiber, and local pull-through of the fiber via a weakened interfacial transition zone (ITZ).

## 4. Analytical Investigation and Discussion

A linear regression analysis was conducted based on a relatively extensive dataset of experimental results.

**Figure 16:**
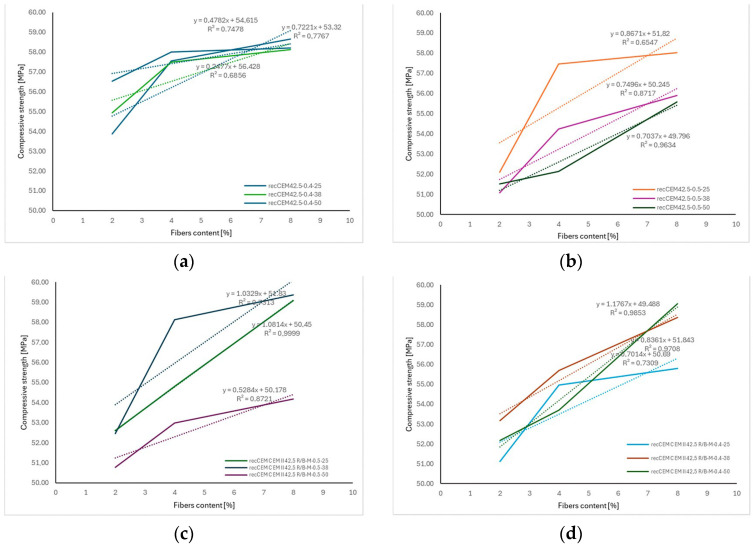
The correlation between fiber length and compressive strength *f_c_* depending: (**a**) recC—CEM I 42.5-0.5 (**b**) recC—CEM I 42.5-0.4 (**c**) recC—CEM II 42.5R/A-V-0.5 (**d**) recC—CEM II 42.5R/A-V-0.4. The dashed lines in the figure represent the trend lines (linear regression) for the obtained test results.

The analysis of the obtained data revealed the clear influence of the length of the applied recycled carbon fibers on the compressive strength of concrete as a function of fiber content (Figure 16). For all tested mixtures, an increase in fiber dosage led to a rise in compressive strength (*f_c_*); however, the intensity of this increase varied depending on the fiber length. Concretes containing fibers with a length of 50 mm exhibited the greatest improvements in compressive strength with increasing fiber content, as reflected by the highest slope coefficients of the trend lines (reaching up to 1.77 MPa per kg/m^3^ of fibers) and very high determination coefficients (R^2^ values close to 0.99), indicating a strong linear relationship.

In the case of 25 mm and 38 mm fibers, a positive effect of increasing fiber content on fc was also observed, although the relationship was weaker and the regression coefficients were lower. These results confirm that both the amount and the length of the fibers play a significant role in enhancing the mechanical properties of concrete, especially in terms of compressive strength.

**Figure 17:**
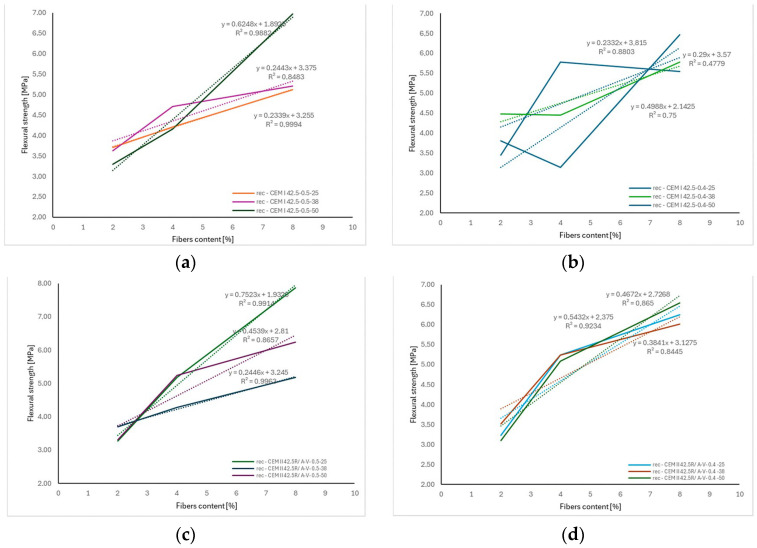
The correlation between fiber length and flexural strength depending: (**a**) recC—CEM I 42.5-0.5 (**b**) recC—CEM I 42.5-0.4 (**c**) recC—CEM II 42.5R/A-V-0.5 (**d**) recC—CEM II 42.5R/A-V-0.4. The dashed lines in the figure represent the trend lines (linear regression) for the obtained test results.

The results presented in Figure 17 clearly indicate a relationship between the length of carbon fibers, their content in the mixture, and the flexural strength of concrete.

For concretes produced with both CEM I 42.5 and CEM II 42.5R/A-V cements, an increase in flexural tensile strength (*f_ct_*) was observed as the fiber content increased. However, the intensity of this increase varied depending on the fiber length. In most cases, the highest regression slope values—up to approximately 0.75 MPa per kg/m^3^ of fibers—were obtained for fibers with a length of 25 mm. This suggests that this length had the most significant impact on enhancing *f_ct_* within the tested range.

In contrast, 50 mm fibers demonstrated a lower rate of increase in *f_ct_* per kg/m^3^ of added fibers. This may be attributed to their tendency to form localized clusters in the mix and cause areas of increased porosity. The high coefficients of determination (R^2^), often exceeding 0.90, confirm a good agreement between the adopted linear models and the experimental data.

These results demonstrate that the combination of fiber length and appropriate dosage is a key factor in shaping the mechanical performance of fiber-reinforced concrete, allowing for effective improvement in flexural strength and potential mitigation of crack propagation.

Analysis of data from numerous literature sources has shown that the flexural strength of concretes with polypropylene (PP) fiber addition increases with the growth of their volumetric content. For example, Mashrei et al. [25] indicated that increasing the PP content from 0% to approximately 0.3% (≈3 kg/m^3^) results in an increase in flexural strength (*f_ct_*) of about 20–30% compared to reference concrete. Studies involving macro PP fibers (length 38–60 mm) in normal-strength concretes (40–50 MPa) at fiber volume contents of 0.5–1.5% confirmed an increase in flexural toughness up to 100% at 1.5% PP content [26].

Regression models from several publications quantitatively describe the relationship between flexural strength and the content of polymer PP fibers. One approach assumes a multiple regression model of the form [27]:

(5) $$f=f_{0}+b_{1}V_{f}+b_{2}l_{f}$$

where *f* is the flexural strength, *V_f_* is the volumetric fiber content, l*_f_* is the fiber length.

In a specific case, the following regression equation was obtained:

(6) $$f=f_{0}+b_{1}X+b_{2}Y{+b}_{3}Z$$

where X is the total fiber content, Y is the PP content, and Z is the glass fiber content.

Moreover, the regression study showed that for every 0.1% increase in PP content (≈1 kg/m^3^), the flexural strength increased by approximately 6–15%, depending on the fiber length (25–50 mm).

**Figure 18:**
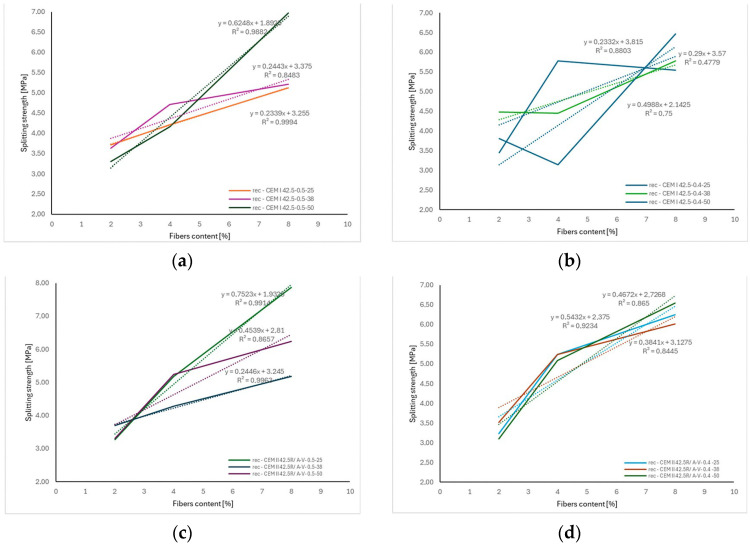
The correlation between fiber length and splitting test depending: (**a**) recC—CEM I 42.5-0.5 (**b**) recC—CEM I 42.5-0.4 (**c**) recC—CEM II 42.5R/A-V-0.5 (**d**) recC—CEM II 42.5R/A-V-0.4. The dashed lines in the figure represent the trend lines (linear regression) for the obtained test results.

**Figure 19:**
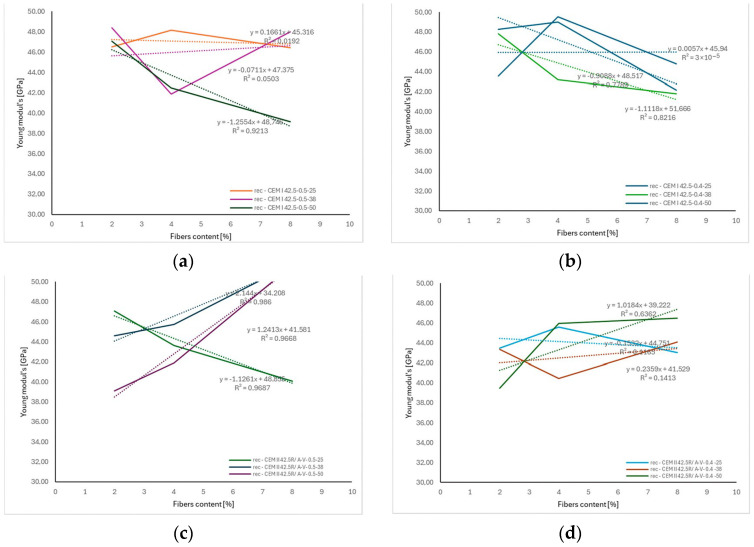
The correlation between fiber length and Young’s modulus depending: (**a**) recC—CEM I 42.5-0.5 (**b**) recC—CEM I 42.5-0.4 (**c**) recC—CEM II 42.5R/A-V-0.5 (**d**) recC—CEM II 42.5R/A-V-0.4. The dashed lines in the figure represent the trend lines (linear regression) for the obtained test results.

Analysis of the results presented in Figure 18 confirms the general trend of increasing splitting tensile strength (*f_cf_*) of concrete with increasing content of recycled carbon fibers. Similar to flexural strength behavior (Figure 19), the strength increments were clearly dependent on fiber length and volumetric content. The obtained regression lines for individual series exhibited high coefficients of determination (R^2^ > 0.8), indicating a strong linear correlation between fiber dosage and the increase in *f_cf_*.

The steepest regression slopes, corresponding to the greatest *f_cf_* gain per kg/m^3^ of added fibers, were observed in concretes reinforced with longer fibers (38 and 50 mm), which can be attributed to more effective crack bridging and better anchorage of fibers in the cement matrix. At the same time, slightly lower coefficients of determination, compared to the flexural strength results, may be due to local variations in fiber distribution within the concrete and the complex stress conditions during the splitting test.

Overall, the observed relationships indicate a similar reinforcement mechanism of concrete under both types of tensile loading, which aligns with literature reports on fiber-reinforced concretes (FRC) [28,29] where linear dependencies between fiber content and tensile strength were also confirmed.

Analysis of the relationships shown in Figure 19 confirms that varying the content of recycled carbon fibers from 0 to 8 kg/m^3^, at different fiber lengths (25, 38, and 50 mm) and in various cement matrix systems (CEM I 42.5 and CEM II 42.5R/A-V), led to significant though inconclusive changes in the elastic modulus (*E_cm_*) of concrete.

In most cases, a trend of slightly decreasing *E_cm_* values with increasing fiber content was observed—particularly evident for mixtures with *w*/*c* = 0.5, CEM I 42.5 cement, and 50 mm fiber length (a reduction of approx. 1.26 GPa per additional kg/m^3^ of fibers, R^2^ = 0.9213). Such behavior aligns with observations from other studies, which have shown that fiber reinforcement may increase matrix porosity or micro-void content, thus lowering the concrete’s elastic modulus [26,30,31,32].

Conversely, for concretes with fly ash cement (CEM II 42.5R/A-V) and a lower water–cement ratio (*w*/*c* = 0.4), opposite tendencies were noted. For fibers of 25 and 38 mm in length, *E_cm_* increased with higher fiber content—by approx. 1.02–1.24 GPa per kg/m^3^ of fibers—with very good fit coefficients (R^2^ > 0.96). This behavior may be linked to a favorable influence of fibers on cement paste microstructure and the reduction in microcracking during early hydration, which partially offsets the introduced voids [33,34,35,36]. However, in some cases (e.g., recC-CEM II 42.5R/A-V-0.4-50), the regression model showed a low coefficient of determination (R^2^ ≈ 0.14), indicating poor fit of the linear model to the experimental data. This may result from the dominant influence of other factors, such as localized fiber clustering, heterogeneous cement matrix microstructure, or random fiber orientation. Similar conclusions were reported in the literature [37,38,39,40], where variation in *E_cm_* for fiber-reinforced concretes was often greater than for compressive or flexural strength.

Fiber-reinforced concrete (FRC) exhibits improved flexural and tensile strength compared to conventional concrete. The formulas used to describe these strengths typically take into account factors such as fiber type, fiber volume fraction *V_f_*, aspect ratio *l_f_*/*d_f_* (length-to-diameter ratio), and the compressive strength of concrete *f_c_*. There is no single universal equation; instead, several empirical and analytical models exist, often tailored to the specific type of fiber and concrete.

In the literature, various formulas can be found for calculating the flexural strength of FRC as a function of compressive strength. The expressions provided in standards are typically formulated as:

- ACI 318R-95 [41]

(7) $$f_{r}=0.62\sqrt{f_{c}}$$

- ACI 363R-92 [42]

(8) $$f_{r}=0.94\sqrt{f_{c}}$$

- Ahmad & Shah [43]

(9) $$f_{r}=0.44\sqrt{f_{c}}$$

- ACI 544.4R-88 (for FRCs in general) [44]

(10) $$f_{r}=k\sqrt{f_{c}}$$

where *k* depends on the type of fibers and their volume fraction (usually 0.6–1.0).

- fib Model Code [45]

(11) $$f_{r}=0.6\sqrt{f_{c}}$$

- JSCE (Japan Society of Civil Engineers, 1983) for concrete with steel fibers [46]

(12) $$f_{r}=0.7\sqrt{f_{c}}$$

Empirical models for different types of fibers:

- Nanni (1989) for polymer fibers in concrete [47]

(13) $$f_{r}=0.55\sqrt{f_{c}}+c_{1}{V\sigma }_{f}$$

where *c*_1_—depends on the type of fiber ~0.3–0.5, *σ_f_*—tensile strength of fibers,

- A. Bentur and S. Mindess (1990) for FRC in general [28]:

(14) $$f_{r}=a+bV_{f}$$

where (typically: $a=0.6\sqrt{f_{c}}$, b ≈ 1–3 for steel fibers in %).

- RILEM TC 162-TDF [48]

(15) $$f_{ct,f}=f_{ct}\left(1+\alpha \frac{l_{f}}{d_{f}}V_{f}\right)$$

where *α*—efficiency factor: 0.2–0.5 for polypropylene (PP) fibers, 0.4–0.8 for PVA fibers (better adhesion),

- Model ACI 544.4R [49]

(16) $$f_{ct,fl}=0.62\sqrt{f_{c}}+kV_{f}\frac{l_{f}}{d_{f}}$$

where *k*—factor depending on fiber type (e.g., 0.5–1.5 for steel fibers).

- Model Swamy and Magat [50]

(17) $$f_{fl}=0.73+8.061V_{f}\frac{l_{f}}{d_{f}}$$

- Model Glinicki [51]

(18) $$f_{fl}=0.97\left(1-V_{f}\right)f_{flc}+3.41V_{f}\frac{l_{f}}{d_{f}}$$

- Model Balaguru and Shah (1992) [52]

(19) $$f_{ct,f}=f_{ct}+\eta V_{f}\sigma_{f}$$

where *η*—fiber efficiency (e.g., 0.3–0.5 for carbon fibers)

**Figure 20:**
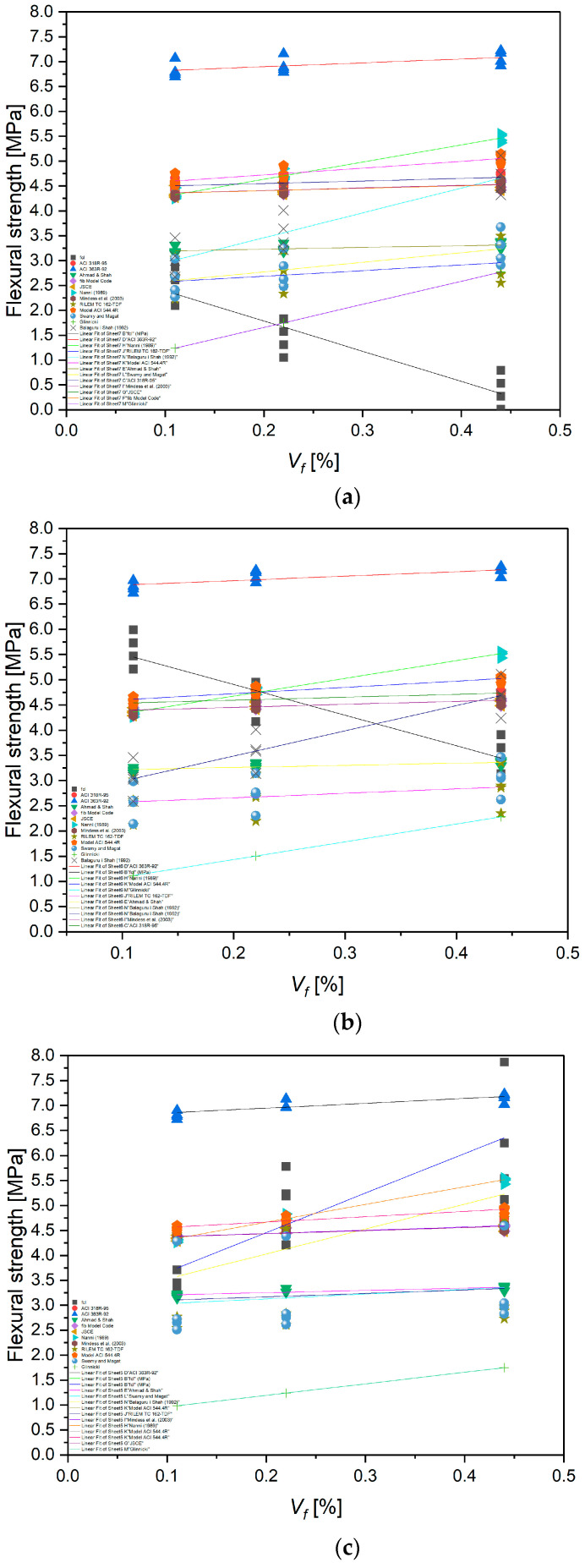
Influence of recycled carbon fiber volume content on flexural strength calculated according to various empirical and semi-empirical models for different fiber lengths: (**a**) 25 mm, (**b**) 38 mm, (**c**) 50 mm [28,41,42,43,44,45,46,47,48,50,51,52].

The analysis of the results calculated using various empirical and semi-empirical models (ACI 318R-95, ACI 363R-92, fib Model Code, JSCE, Nanni, Mindess et al., RILEM TC 162-TDF, Swamy and Magat, and Balaguru and Shah), presented in Figure 20 and Table 3, demonstrated, demonstrated a clear influence of both the fiber volume fraction (*V_f_*) and their aspect ratio (*l_f_*/*d_f_*) on the predicted flexural strength of fiber-reinforced concrete. Increasing the fiber content from 0.1% to 0.4% and the aspect ratio from approximately 28 to over 57 resulted in an increase in the calculated flexural strength values by approximately 20–40%, depending on the model applied. The most sensitive models to these parameters were those by Nanni and RILEM TC 162-TDF, which directly account for the fiber slenderness and their effectiveness in bridging cracks. These findings are consistent with the fiber reinforcement mechanism in cement composites—longer and more slender fibers, at higher volume fractions, more effectively hinder crack propagation and enhance the concrete’s ability to transfer stress in the post-cracking phase. Similar relationships have been reported by Mindess et al. [28] and Balaguru and Shah [52].

**Figure 21:**
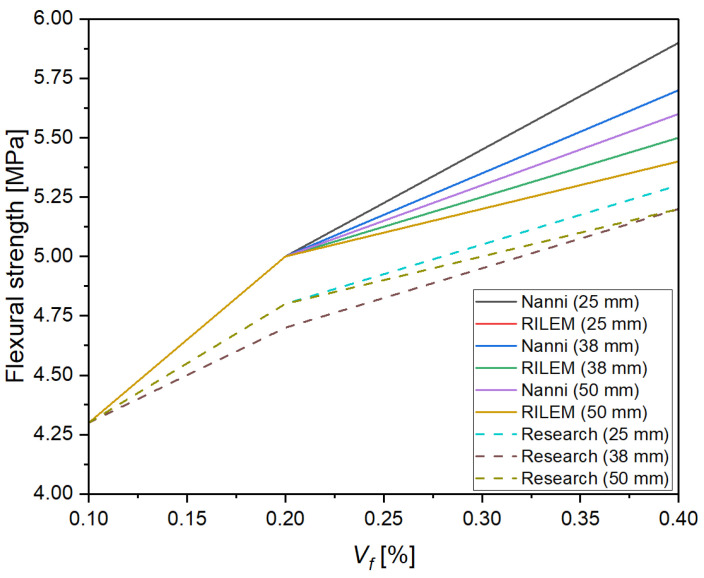
Comparison of Models and Experimental Results for Different Fiber Lengths.

The analysis of the results presented in Figure 21 demonstrated that an increase in the fiber volume fraction *V_f_* from 0.1% to 0.4% leads to a systematic increase in the flexural strength of concrete, regardless of the fiber length used. The highest values were recorded for fibers with a length of 25 mm, where at *V_f_* = 0.4%, the flexural strength increased to approximately 5.5 MPa, representing an increase of nearly 28% compared to the mixture with *V_f_* = 0.1%. Similar trends were observed for fibers with lengths of 38 mm and 50 mm, although with a slightly lower growth rate. This may be related to fiber agglomeration or limitations stemming from the mixing process and the uniformity of fiber distribution in the cement matrix. These results indicate that within the analyzed parameter range, fiber length plays a lesser role than fiber volume content, which is consistent with findings in the literature, including works by Mindess et al. and the RILEM TC 162-TDF model, both of which emphasize the key role of fiber quantity in crack bridging capacity and enhancing the post-cracking behavior of concrete.

Figure 21 presents a comparison of the flexural strength values of concrete reinforced with recycled carbon fibers obtained from experimental studies and those predicted by the RILEM TC 162-TDF [48] and Nanni (1989) [47] models for three fiber lengths (25, 38, and 50 mm) as a function of fiber volume content *V_f_*. The analysis showed that with an increase in *V_f_* from 0.1% to 0.4%, flexural strength increased consistently in both experimental measurements and model predictions. The highest strength gains were recorded for mixes with 25 mm fibers, likely due to better dispersion of shorter fibers in the cement matrix and a lower tendency for agglomeration.

Both the RILEM TC 162-TDF [48] and Nanni (1989) [47] models captured the overall trend of increasing strength, but at higher fiber contents (0.4%), the Nanni model tended to overestimate the results, particularly for longer fibers (38 and 50 mm). In contrast, the RILEM model predictions were more consistent with the experimental results across the entire parameter range. These findings highlight the need for cautious application of general empirical models when predicting the flexural strength of concrete reinforced with recycled carbon fibers, and they emphasize the importance of calibrating these models based on laboratory results tailored to specific fiber and matrix systems.

**Figure 22:**
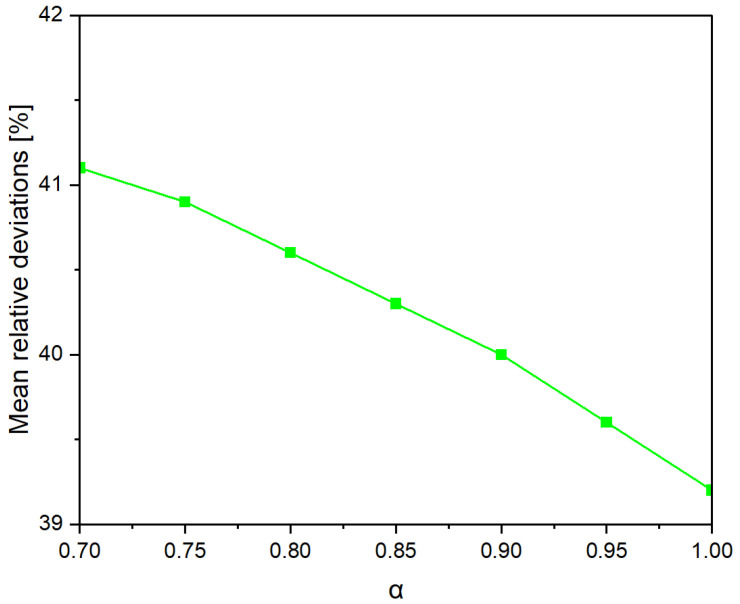
Effect of α on the Fit of the RILEM TC 162-TDF Model to Experimental Results.

To verify the suitability of empirical models for predicting the flexural strength of concrete reinforced with recycled carbon fibers, the predicted results from the RILEM TC 162-TDF [48] and Nanni (1989) [47] formulas were calibrated against the experimental values. The calculated mean relative deviations showed that for the tested mixtures, the RILEM model with a classic efficiency coefficient of α = 0.8 slightly underestimated the strength (deviations of about 4–7%), whereas the Nanni model, at higher fiber contents (0.4%), overestimated the values by approximately 8–10%. Further analysis revealed that increasing the efficiency coefficient in the RILEM model to α = 0.9 provided significantly better alignment with the experimental results (mean deviations < 3%). This suggests that for high-stiffness, well-bonded recycled carbon fibers, a higher efficiency coefficient is recommended. The proposed correction allows for a more realistic representation of crack-bridging mechanisms in concrete reinforced with recycled carbon fibers and can be used in future design analyses (Figure 22).

It can be observed that the minimum error (~2–3%) occurs for α close to 0.9, confirming that for concrete with recycled carbon fibers, a higher α value than the typical 0.8 is advisable. The following modified formula is proposed by the author:

(20) $$f_{ct,f}=f_{ct}\left(1+0.9\frac{l_{f}}{d_{f}}V_{f}\right)$$

The conducted research and analyses demonstrated that the fiber volume fraction *V_f_* plays a key role in enhancing the flexural strength of concrete reinforced with recycled carbon fibers. The locally derived regression model confirmed the significant influence of *V_f_*; an increase from 0.1% to 0.4% led to a strength gain of approximately 40–50%. Within the analyzed parameter range, it was observed that fiber slenderness *l_f_*/*d_f_* had only a minor, and in this case even slightly negative, effect on strength. This may be attributed to the tendency of more slender fibers to agglomerate and reduce the uniformity of the mix, which limits their effectiveness in crack bridging. The obtained model indicates that within the studied range, the fiber volume fraction had a greater impact on concrete strength than either fiber length or aspect ratio.

**Figure 23:**
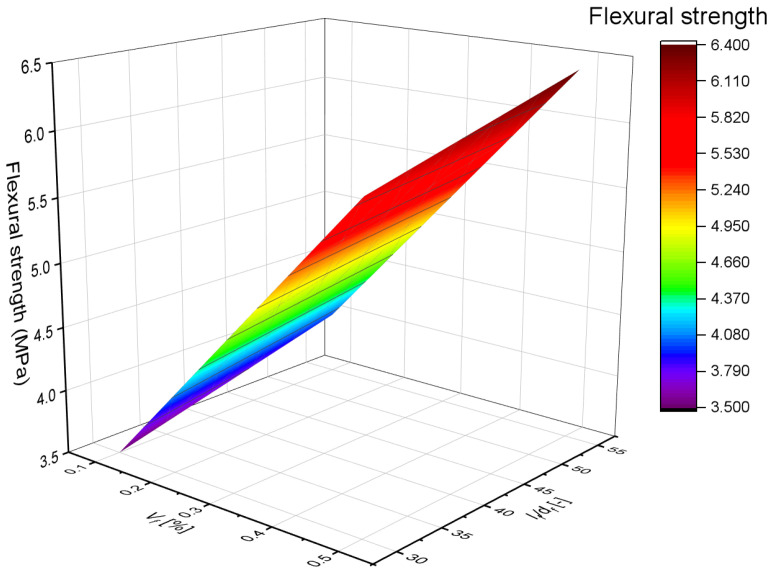
Effect of Volume Fraction and Slenderness on Concrete Strength.

The three-dimensional analysis (Figure 23) enabled assessment of the simultaneous influence of fiber volume fraction *V_f_* and slenderness *l_f_*/*d_f_* on the flexural strength of concrete reinforced with recycled carbon fibers. The results clearly showed that the dominant factor contributing to strength enhancement is the fiber volume fraction, with an increase from 0.1% to 0.4% resulting in a significant strength gain of approximately 40–50%. In contrast, fiber slenderness had a relatively minor and slightly negative effect in this series of tests, which may be related to local clustering of more slender fibers and difficulties in their uniform dispersion within the concrete matrix. The surface model based on experimental data confirms the crucial role of fiber volume in post-cracking stress transfer mechanisms and suggests that further increases in fiber slenderness without ensuring adequate mix uniformity may not yield additional benefits in terms of strength.

The conducted multiple regression analysis, based on all experimental results and the literature data compiled in Table 4, allowed the development of a local empirical model describing the relationship between the flexural strength of concrete reinforced with recycled carbon fibers and its compressive strength, fiber volume fraction, and fiber slenderness.

To estimate the flexural tensile strength of concrete reinforced with recycled carbon fibers, a multiple linear regression model was developed. The following independent variables were used: *f_c_*—compressive strength of concrete (MPa), *l_f_*/*d_f_*—fiber slenderness (length-to-diameter ratio), *V_f_* (%)—fiber volume fraction.

Additionally, the interaction term *Vf (l_f_*/*d_f_)* was tested to evaluate the nonlinear influence of fiber geometry and dosage.

The model was expressed as:

(21) $$f_{ct}=k_{1}f_{ck}+k_{2}V_{f}+k_{3}\frac{l_{f}}{d_{f}}+k_{4}$$

where *k*_1_ = 0.0801, *k*_2_ = 4.6847, *k*_3_ = 0.0372, *k*_4_ = −2.657—coefficients determined experimentally.

The model demonstrates a very good fit: R^2^ = 0.787, adjusted R^2^ = 0.773, and all main predictors are statistically significant (*p* < 0.05), except for the interaction term *V_f_* (*l_f_*/*d_f_*), which has a *p*-value of 0.131 and was therefore excluded from further consideration.

**Figure 24:**
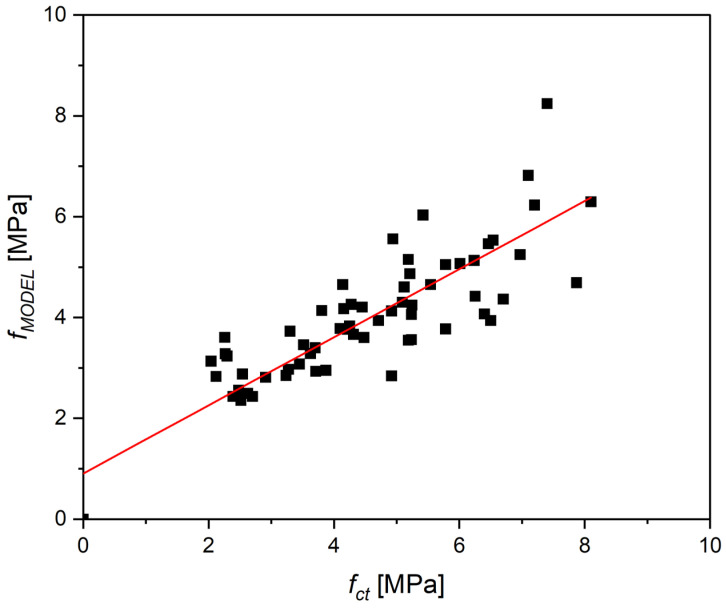
Relationship between experimental flexural tensile strength values (*f_ct_*) and those predicted by the model (*f_MODEL_*).

A comparison plot of the predicted values (*f_MODEL_*) versus the actual flexural tensile strengths (*f_ct_*) is presented in Figure 24. A good agreement between the model and the experimental data is observed, especially in the range of approximately 2 to 6 MPa. Most data points are concentrated near the regression line, indicating accurate modeling of the overall trend. At higher strength levels (above 6 MPa), a slightly greater data dispersion was noted, suggesting a need for further calibration. Nevertheless, the obtained fit is sufficiently accurate for analytical and engineering purposes within the considered parameter range.

The model was calibrated based on the authors’ own laboratory tests, covering a range up to *V_f_* = 1% and *l_f_*/*d_f_* ≤ 60. Therefore, its applicability should be limited to similar parameter ranges. These boundaries result from observed deviations that increase markedly beyond 1% fiber content and for very long fibers (*l_f_*/*d_f_* > 60).

To enhance the generalizability and accuracy of the model, it is recommended to gather a larger dataset through extended experimental campaigns. Future research should include a broader range of fiber lengths and geometries, as well as diverse concrete matrices (e.g., incorporating pozzolanic additives, fly ash, or recycled aggregates). Additionally, the model could be expanded with nonlinear terms, interaction effects, or parameters related to fiber–matrix bonding. These enhancements would help better capture the behavior of fiber-reinforced concrete mixes with high fiber content and support the optimization of composite design in terms of required mechanical performance.

## 5. Conclusions

This study explored the feasibility and performance of incorporating recycled carbon fibers recovered from decommissioned wind turbine blades into fiber-reinforced concrete (FRC) as a sustainable reinforcement strategy. Based on extensive experimental and analytical investigations, the following conclusions can be drawn:

1. Recycled carbon fibers significantly enhanced the flexural and splitting tensile strength of concrete. The highest fiber dosage (8 kg/m^3^) led to flexural strength gains exceeding 60% and splitting tensile strength gains over 100% relative to plain concrete. The improvement was attributed to the fibers’ efficient crack-bridging behavior and high tensile strength.
2. Compressive strength was mainly governed by the water-to-cement (*w*/*c*) ratio rather than fiber dosage or length. The inclusion of fibers had a marginal effect on compressive strength and showed mixed trends for Modulus of elasticity (*E_cm_*)—with slight reductions in some mixes (due to fiber clustering and increased porosity) and marginal increases in others, particularly those containing CEM II blended cement.
3. Shorter fibers (25 mm) were more effective in increasing flexural strength, while longer fibers (50 mm) provided greater gains in splitting tensile strength. These differences reflect the interaction between fiber anchorage, crack width, and the distribution uniformity of fibers.
4. Increasing fiber content significantly reduced the workability of the concrete mix, particularly at higher dosages and longer fiber lengths. This highlights the need for careful mix design adjustments (e.g., use of superplasticizers) to maintain acceptable consistency levels in practical applications.
5. Fiber inclusion led to a slight reduction in concrete density due to the lower specific weight of carbon fibers and potential for increased air content. Microscopy revealed relatively uniform fiber dispersion at lower dosages and localized clustering at higher contents, with the dominant failure mechanism being fiber pull-out—beneficial for energy dissipation and post-cracking performance.
6. Empirical and standard-based models (e.g., RILEM TC 162-TDF, Nanni, Ahmad & Shah) generally underestimated the flexural strength of recycled-carbon-fiber-reinforced concrete. Calibration of these models (e.g., adjusting the α-factor in RILEM from 0.8 to 0.9) yielded better alignment with experimental data. A new regression model was developed that effectively predicted flexural strength based on compressive strength, fiber volume fraction, and aspect ratio.
7. The use of recycled carbon fibers aligns with circular economy principles, offering a viable solution for composite waste management while enhancing concrete performance. The findings support the implementation of this reinforcement strategy in structural and non-structural applications, especially where enhanced ductility and crack resistance are desired.

Future research on recycled carbon-fiber-reinforced concrete should focus on optimizing fiber processing methods to improve fiber cleanliness, uniformity, and dispersion within the cementitious matrix. Further investigation into surface modification techniques for recycled fibers may enhance fiber–matrix bonding, leading to better mechanical performance. Expanding the range of fiber dosages, lengths, and geometries, as well as combining recycled carbon fibers with other reinforcement types (hybrid reinforcement) could yield synergistic effects.

## Figures and Tables

**Table 1:** Mixture proportions to produce one cubic meter of concrete.

Mixture Proportions	*w*/*c* = 0.5	*w*/*c* = 0.4
CEM I 42.5R or CEM II 42.5R/A-V [kg]	320	320
Water [kg]	160	128
Sand 0.125–2 mm [kg]	732	742
Aggregate 2/16 [kg]	1203	1203
Sika Sikacem Superplast [kg]	3.2	6.4

**Table 2:** Results from uniaxial tensile strength tests.

1	*F_max_*	*R_m_*	Relative Elongation at *R_m_*	Modulus of Elasticity	Elongation at Tensile Strength Point	True Stress at Maximum Displacement During Tension
	[N]	[MPa]	[%]	[MPa]	[%]	[MPa]
1	770.092	1283.486	0.46	302,642.10	0.023	546.294
2	656.093	1093.488	0.38	331,850.89	0.377	698.943
3	661.662	1102.770	0.36	310,719.00	0.007	298.007
4	716.602	1194.337	0.38	322,984.50	0.006	258.448
average	701.112	1168.520	0.95	317,049.12	0.103	450.423

**Table 3:** The flexural tensile strength of FRC calculated using empirical formulas and resulting from the tests carried out.

Type of Cement	*l_f_*	*V_f_*	*l_f_*/*d_f_*	*f_c_*	*f_ct_*	ACI 318R-95 [41]	ACI 363R-92 [42]	Ahmad & Shah [43]	Fib Model Code [45]	JSCE [46]	Nanni (1989) [47]	A. Bentur and S. Mindess (1990) [28]	RILEM TC 162-TDF [48]	ACI 544.4R [49]	Swamy and Magat [50]	Glinicki [51]	Balaguru and Shah (1992) [52]
[-]	[mm]	[%]	[-]	[MPa]	[MPa]	[MPa]	[MPa]	[MPa]	[MPa]	[MPa]	[MPa]	[MPa]	[MPa]	[MPa]	[MPa]	[MPa]	[MPa]
*w*/*c* = 0.5																	
CEM I 42.5	25	0.1	28.7	52.1	3.7	4.5	6.8	3.2	4.3	4.3	4.3	4.3	2.7	4.5	2.7	1.0	3.2
CEM I 42.5	25	0.2	28.7	57.5	4.2	4.7	7.1	3.3	4.5	4.5	4.8	4.6	2.8	4.8	2.8	1.2	3.7
CEM I 42.5	25	0.4	28.7	58.0	5.1	4.7	7.2	3.4	4.6	4.6	5.5	4.6	2.9	4.9	3.0	1.7	4.8
CEM I 42.5	38	0.1	43.7	51.1	3.6	4.4	6.7	3.1	4.3	4.3	4.3	4.3	3.0	4.5	3.0	1.1	3.5
CEM I 42.5	38	0.2	43.7	54.2	4.7	4.6	6.9	3.2	4.4	4.4	4.7	4.4	3.1	4.7	3.1	1.5	4.0
CEM I 42.5	38	0.4	43.7	55.9	5.2	4.6	7.0	3.3	4.5	4.5	5.4	4.5	3.4	4.9	3.5	2.3	5.1
CEM I 42.5	50	0.1	57.5	51.5	3.3	4.5	6.7	3.2	4.3	4.3	4.3	4.3	3.1	4.5	3.0	1.2	3.5
CEM I 42.5	50	0.2	57.5	52.1	4.2	4.5	6.8	3.2	4.3	4.3	4.6	4.3	3.2	4.7	3.2	1.7	4.0
CEM I 42.5	50	0.4	57.5	55.6	7.0	4.6	7.0	3.3	4.5	4.5	5.4	4.5	3.5	5.0	3.7	2.8	5.1
*w*/*c* = 0.4																	
CEM I 42.5	25	0.1	28.7	53.9	3.5	4.6	6.9	3.2	4.4	4.4	4.4	4.4	2.5	4.6	2.5	1.0	3.0
CEM I 42.5	25	0.2	28.7	57.6	5.8	4.7	7.1	3.3	4.6	4.6	4.8	4.6	2.6	4.8	2.6	1.2	3.6
CEM I 42.5	25	0.4	28.7	58.7	5.5	4.7	7.2	3.4	4.6	4.6	5.5	4.6	2.7	4.9	2.8	1.7	4.7
CEM I 42.5	38	0.1	43.7	54.9	4.5	4.6	7.0	3.3	4.4	4.4	4.4	4.5	2.6	4.7	2.6	1.1	3.0
CEM I 42.5	38	0.2	43.7	57.5	4.5	4.7	7.1	3.3	4.5	4.5	4.8	4.6	2.7	4.8	2.7	1.5	3.6
CEM I 42.5	38	0.4	43.7	58.1	5.8	4.7	7.2	3.4	4.6	4.6	5.5	4.6	2.9	5.0	3.1	2.3	4.7
CEM I 42.5	50	0.1	57.5	56.5	3.8	4.7	7.1	3.3	4.5	4.5	4.5	4.5	2.2	4.8	2.3	1.2	2.7
CEM I 42.5	50	0.2	57.5	58.0	3.1	4.7	7.2	3.4	4.6	4.6	4.8	4.6	2.3	4.9	2.5	1.7	3.2
CEM I 42.5	50	0.4	57.5	58.2	6.5	4.7	7.2	3.4	4.6	4.6	5.5	4.6	2.5	5.1	2.9	2.8	4.3
*w*/*c* = 0.5																	
CEM II 42.5 R/B-M (S-V)	25	0.1	28.7	52.6	3.3	4.5	6.8	3.2	4.4	4.4	4.3	4.4	2.8	4.5	2.7	1.0	3.3
CEM II 42.5 R/B-M (S-V)	25	0.2	28.7	54.8	5.2	4.6	7.0	3.3	4.4	4.4	4.7	4.4	2.8	4.7	2.8	1.2	3.8
CEM II 42.5 R/B-M (S-V)	25	0.4	28.7	59.1	7.9	4.8	7.2	3.4	4.6	4.6	5.5	4.6	3.0	5.0	3.0	1.7	4.9
CEM II 42.5 R/B-M (S-V)	38	0.1	43.7	52.5	3.7	4.5	6.8	3.2	4.3	4.3	4.3	4.3	2.6	4.6	2.6	1.1	3.1
CEM II 42.5 R/B-M (S-V)	38	0.2	43.7	58.1	4.3	4.7	7.2	3.4	4.6	4.6	4.9	4.6	2.7	4.9	2.8	1.5	3.6
CEM II 42.5 R/B-M (S-V)	38	0.4	43.7	59.4	5.2	4.8	7.2	3.4	4.6	4.6	5.6	4.6	2.9	5.1	3.1	2.3	4.7
CEM II 42.5 R/B-M (S-V)	50	0.1	57.5	50.8	3.3	4.4	6.7	3.1	4.3	4.3	4.2	4.3	2.7	4.5	2.7	1.2	3.1
CEM II 42.5 R/B-M (S-V)	50	0.2	57.5	53.0	5.2	4.5	6.8	3.2	4.4	4.4	4.7	4.4	2.8	4.7	2.9	1.7	3.6
CEM II 42.5 R/B-M (S-V)	50	0.4	57.5	54.2	6.2	4.6	6.9	3.2	4.4	4.4	5.4	4.4	3.1	4.9	3.3	2.8	4.7
*w*/*c* = 0.4																	
CEM II 42.5 R/B-M (S-V)	25	0.1	28.7	51.1	3.2	4.4	6.7	3.1	4.3	4.3	4.3	4.3	4.4	4.5	4.3	1.0	4.9
CEM II 42.5 R/B-M (S-V)	25	0.2	28.7	55.0	5.2	4.6	7.0	3.3	4.4	4.4	4.7	4.5	4.5	4.7	4.4	1.2	5.4
CEM II 42.5 R/B-M (S-V)	25	0.4	28.7	55.8	6.3	4.6	7.0	3.3	4.5	4.5	5.4	4.5	4.7	4.8	4.6	1.7	6.5
CEM II 42.5 R/B-M (S-V)	38	0.1	43.7	53.2	3.5	4.5	6.9	3.2	4.4	4.4	4.3	4.4	2.1	4.6	2.1	1.1	2.6
CEM II 42.5 R/B-M (S-V)	38	0.2	43.7	55.7	5.2	4.6	7.0	3.3	4.5	4.5	4.8	4.5	2.2	4.8	2.3	1.5	3.1
CEM II 42.5 R/B-M (S-V)	38	0.4	43.7	58.4	6.0	4.7	7.2	3.4	4.6	4.6	5.5	4.6	2.4	5.0	2.6	2.3	4.2
CEM II 42.5 R/B-M (S-V)	50	0.1	57.5	52.2	3.1	4.5	6.8	3.2	4.3	4.3	4.3	4.3	2.4	4.6	2.4	1.2	2.8
CEM II 42.5 R/B-M (S-V)	50	0.2	57.5	53.7	5.1	4.5	6.9	3.2	4.4	4.4	4.7	4.4	2.5	4.7	2.6	1.7	3.4
CEM II 42.5 R/B-M (S-V)	50	0.4	57.5	59.1	6.5	4.8	7.2	3.4	4.6	4.6	5.5	4.6	2.7	5.1	3.0	2.8	4.5

**Table 4:** The flexural tensile strength of FRC calculated using empirical formulas and resulting from the test (✔—in the model, ✖—not in the model).

Ref.	*l_f_*/*d_f_*	*V_f_*	*f_ct_*	Comments
	[-]	[%]	[MPa]	
own research	29	0	2.63	✔
	29	0.11	3.71	
	29	0.22	4.21	
	29	0.44	5.12	
	29	0	2.48	
	29	0.11	3.45	
	29	0.22	5.78	
	29	0.44	5.54	
	29	0	2.70	
	29	0.11	3.28	
	29	0.22	5.19	
	29	0.44	7.87	
	29	0	2.52	
	29	0.11	3.24	
	29	0.22	5.24	
	29	0.44	6.25	
	44	0	2.91	
	44	0.11	3.63	
	44	0.22	4.71	
	44	0.44	5.21	
	44	0	2.12	
	44	0.11	4.48	
	44	0.22	4.45	
	44	0.44	5.78	
	44	0	2.54	
	44	0.11	3.70	
	44	0.22	4.28	
	44	0.44	5.19	
	44	0	3.87	
	44	0.11	3.52	
	44	0.22	5.24	
	44	0.44	6.01	
	57	0	2.30	
	57	0.11	3.30	
	57	0.22	4.16	
	57	0.44	6.97	
	57	0	2.26	
	57	0.11	3.81	
	57	0.22	4.14	
	57	0.44	6.47	
	57	0	2.04	
	57	0.11	4.31	
	57	0.22	5.25	
	57	0.44	6.24	
	57	0	2.27	
	57	0.11	4.10	
	57	0.22	5.09	
	57	0.44	6.54	
[14]	50	0	4.46	✔
	50	0.5	4.92	✔
	50	1	5.42	✔
	50	1.5	5.39	✖
[53]	0	0	2.62	✔
	25	0.5	2.61	✔
	25	1	2.6	✔
	25	1.5	2.54	✖
	0	0	2.62	✔
	38	0.5	2.78	✔
	38	1	2.8	✔
	38	1.5	2.64	✖
	0	0	2.62	✔
	50	0.5	2.93	✔
	50	1	2.78	✔
	50	1.5	2.68	✖
[54]	0	0	5.97	✔
	40	1	8.1	✔
[55]	0	0	4.46	✔
	18	0.5	4.92	✔
	18	1	5.42	✔
	18	1.5	5.39	✖
	0	0	4.16	✔
	18	0.5	4.25	✔
	18	1	4.94	✔
	18	1.5	5.52	✖
[56]	0	0	3.72	✔
	0	0	3.54	✔
	0	0	4.38	✔
	0	0	3.35	✔
	20	0.5	4.92	✔
	20	1.5	4.24	✖
	20	2.5	4.53	✖
	20	3.5	5.12	✖
[57]	0	0	5.4	✔
	10	0.5	6.5	✔
	10	1	7.2	✔
	10	1.5	6.8	✖
	10	2	6.4	✖
	20	0.5	6.4	✔
	20	1.5	7.4	✔
	20	2	6.8	✖
	30	0.5	6.7	✔
	30	1	7.1	✔
	30	1.5	6.7	✖
	30	2	6.4	✖
[58]	0	0	2.2	✖
	95	0.25	4.3	✖
	95	0.375	4.6	✖
	95	0.5	6.2	✖
[59]	0	0	2	✖
	714	0.2	4.72	✖
	714	0.4	4.38	✖
	714	0.6	4.26	✖
	714	0.8	4.11	✖
[60]	0	0	11.7	✖
	65	1.5	12.9	✖
	1455	1.5	10.8	✖
	1764	1.5	9.83	✖
	224	1.5	10.7	✖

## Data Availability

The original contributions presented in this study are included in the article. Further inquiries can be directed to the corresponding author.

## References

[1] Arslan G., Keskin R.S.O., Ulusoy S. (2017). An experimental study on the shear strength of SFRC beams without stirrups. J. Theor. Appl. Mech..

[2] Muthukumarana T.V., Arachchi M.A.V.H.M., Somarathna H.M.C.C., Raman S.N. (2023). A review on the variation of mechanical properties of carbon fibre-reinforced concrete. Constr. Build. Mater..

[3] Wei A., Tan M.Y., Koay Y.-C., Hu X., Al-Ameri R. (2021). Effect of carbon fiber waste on steel corrosion of reinforced concrete structures exposed to the marine environment. J. Clean. Prod..

[4] Cardoso D.C.T., Pereira G.B.S., Silva F.A., Filho J.J.H.S., Pereira E.V. (2019). Influence of steel fibers on the flexural behavior of RC beams with low reinforcing ratios: Analytical and experimental investigation. Compos. Struct..

[5] Carmona J.R., Ruiz G. (2014). Bond and size effect on the shear capacity of RC beams without stirrups. Eng. Struct..

[6] Artemenko S.E. (2003). Polymer Composite Materials Made from Carbon, Basalt, and Glass Fibres. Structure and Properties. Fibre Chem..

[7] Baumgaertel E., Marx S. (2023). The Recycling of Carbon Components and the Reuse of Carbon Fibers for Concrete Reinforcements. Appl. Sci..

[8] Zhou Z., Zhao B., Lone U.A., Fan Y. (2024). Experimental study on mechanical properties of shredded prepreg carbon cloth waste fiber reinforced concrete. J. Clean. Prod..

[9] Waqar A., Khan M.B., Afzal M.T., Radu D., Gălăţanu T., Cazacu C.E., Dodo Y., Althoey F., Almujibah H.R. (2024). Investigating the synergistic effects of carbon fiber and silica fume on concrete strength and eco-efficiency. Case Stud. Constr. Mater..

[10] Ge L., Li X., Feng H., Xu C., Lu Y., Chen B., Li D., Xu C. (2023). Analysis of the pyrolysis process, kinetics and products of the base components of waste wind turbine blades (epoxy resin and carbon fiber). J. Anal. Appl. Pyrolysis.

[11] Lin T.A., Chuang Y.-C., Lin J.-Y., Lin M.-C., Lou C.-W., Lin J.-H. (2019). Weaving carbon fiber/recycled polypropylene selvages to reinforce the polymer-based protective composite fabrics: Manufacturing techniques and electromagnetic shielding effectiveness. Polym. Compos..

[12] Xiong C., Li Q., Lan T., Li H., Long W., Xing F. (2021). Sustainable use of recycled carbon fiber reinforced polymer and crumb rubber in concrete: Mechanical properties and ecological evaluation. J. Clean. Prod..

[13] Li Y.-F., Li J.Y., Ramanathan G.K., Chang S.M., Shen M.Y., Tsai Y.K., Huang C.H. (2021). An Experimental Study on Mechanical Behaviors of Carbon Fiber and Microwave-Assisted Pyrolysis Recycled Carbon Fiber-Reinforced Concrete. Sustainability.

[14] Dai L., Hu X., Zhao C., Zhou H., Zhang Z., Wang Y., Ma S., Liu X., Li X., Shu X. (2024). Machine learning constructs the microstructure and mechanical properties that accelerate the development of CFRP pyrolysis for carbon-fiber recycling. Waste Manag..

[15] Baskar S., Subbiah G., Guntaj J., Kumar M., Kaliappan N. (2025). Development and characterization of an epoxy matrix composite reinforced with Al_2_O_3_ embedded banyan fibers for secondary structural applications. Results Eng..

[16] Valente M., Sambucci M., Rossitti I., Abruzzese S., Sergi C., Sarasini F., Tirillò J. (2023). Carbon-Fiber-Recycling Strategies: A Secondary Waste Stream Used for PA6,6 Thermoplastic Composite Applications. Materials.

[17] (2012). ACI 440.3R-12 Guide Test Methods for Fiber-Reinforced Polymer (FRP) Composites for Reinforcing or Strengthening Concrete Structures.

[18] ACI Committee 440 (2004). Guide Test Methods for Fiber Reinforced Polymers (FRPs) for Reinforcing or Strengthening Concrete Structures.

[19] (2019). Testing Fresh Concrete Sampling and Common Apparatus.

[20] (2019). Testing Hardened Concrete—Part 7: Density of Hardened Concrete.

[21] (2019). Testing Hardened Concrete—Part 3: Compressive Strength of Test Specimens.

[22] (2019). Testing Hardened Concrete—Part 5: Flexural Strength of Test Specimens.

[23] (2010). Testing Hardened Concrete—Part 6: Tensile Splitting Strength of Test Specimens.

[24] (2014). Testing Hardened Concrete—Part 13: Determination of Secant Modulus of Elasticity in Compression.

[25] Mashrei M., Sultan A., Mahdi A.M. (2018). Effects of polypropylene fibers on compressive and flexural strength of concrete material. Int. J. Civ. Eng. Technol..

[26] Blazy J., Blazy R. (2021). Polypropylene fiber reinforced concrete and its application in creating architectural forms of public spaces. Case Stud. Constr. Mater..

[27] Balasubramanian N., Subasini Y., Shanmugam P. (2022). Modelling and Prediction of Strength for Polypropylene Fiber Reinforced Concrete. Recent Developments in Sustainable Infrastructure (ICRDSI-2020)—Structure and Construction Management: Conference Proceedings from ICRDSI-2020.

[28] Bentur A., Mindess S. (1990). Fibre Reinforced Cementitious Composites. Elsevier Applied Science.

[29] Banthia N., Gupta R. (2006). Influence of polypropylene fiber geometry on plastic shrinkage cracking in concrete. Cem. Concr. Res..

[30] van den Heever M., Plessis A.D., Kruger J., van Zijl G. (2022). Evaluating the effects of porosity on the mechanical properties of extrusion-based 3D printed concrete. Cem. Concr. Res..

[31] Zhang G., Yang Z., Yan Y., Wang M., Wu L., Lei H., Gu Y. (2021). Experimental and Theoretical Prediction Model Research on Concrete Elastic Modulus Influenced by Aggregate Gradation and Porosity. Sustainability.

[32] Alshahrani A., Kulasegaram S., Kundu A. (2023). Elastic modulus of self-compacting fibre reinforced concrete: Experimental approach and multi-scale simulation. Case Stud. Constr. Mater..

[33] Zhang X., Pel L., Smeulders D. (2024). Influence of water-soluble leachates from natural fibers on the hydration and microstructure of cement paste studied by nuclear magnetic resonance. Cem. Concr. Res..

[34] Wu J., Ding Q., Yang W., Wang L., Wang H. (2021). Influence of Submicron Fibrillated Cellulose Fibers from Cotton on Hydration and Microstructure of Portland Cement Paste. Molecules.

[35] Azevedo A., De Matos P., Marvila M., Sakata R., Silvestro L., Gleize P., Brito J.D. (2021). Rheology, Hydration, and Microstructure of Portland Cement Pastes Produced with Ground Açaí Fibers. Appl. Sci..

[36] Tran N.P., Gunasekara C., Law D.W., Houshyar S., Setunge S. (2022). Microstructural characterisation of cementitious composite incorporating polymeric fibre: A comprehensive review. Constr. Build. Mater..

[37] Wang Y., Pan L., Niu W., Li K., Guo K. (2023). Variability Analysis of the Hysteretic Behavior of Fiber-Reinforced Polymer (FRP)-Confined Concrete Columns Based on a Secondary Development Model. Buildings.

[38] Kahanji C., Ali F., Nadj A. (2017). Structural performance of ultra-high-performance fiber-reinforced concrete beams. Struct. Concr..

[39] Thomas J., Ramaswamy A. (2007). Mechanical Properties of Steel Fiber-Reinforced Concrete. J. Mater. Civ. Eng..

[40] Khalel H.H., Khan M., Starr A., Sadawi N., Mohamed O.A., Khalil A., Esaker M. (2025). Parametric study for optimizing fiber-reinforced concrete properties. Struct. Concr..

[41] (1995). Building Code Requirements for Structural Concrete (ACI 318-95) and Commentary (ACI 318R-95).

[42] ACI 363R-92; Report on High-Strength Concrete (Reapproved 1997). https://www.concrete.org/publications/internationalconcreteabstractsportal/m/details/id/5194.

[43] Ahmad S.H., Shah S.P. (1985). Structural properties of high-strength concrete and its implications for pre-cast prestressed concrete. PCI J..

[44] (2009). 4R-88; Design Considerations for Steel Fiber Reinforced Concrete. https://www.concrete.org/store/productdetail.aspx?ItemID=544488&Format=DOWNLOAD&Language=English&Units=US_AND_METRIC.

[45] Fib Model Code 2010 (2013). Fib Model Code for Concrete Structures 2010.

[46] Japan Society of Civil Engineers (JSCE) (2007). Standard Specifications for Concrete Structures—2007: Design.

[47] Nanni A. (1993). Flexural Behavior and Design of RC Members Using FRP Reinforcement. J. Struct. Eng..

[48] Vandewalle L., Nemegeer D., Balazs G.L., Barr B., Bartos P., Banthia N., Brandt A.M., Criswell M., Denarie F., Di Prisco M. (2001). RILEM TC 162-TDF: Test and design methods for steel fibre reinforced concrete—Uni-axial tension test for steel fibre reinforced concrete. Mater. Struct..

[49] (2018). 4R-18; Guide for Design with Fiber-Reinforced Concrete. https://www.scribd.com/document/807774534/ACI-544-4R-18-Standard.

[50] Swamy R.N., Mangat P.S. (1974). Influence of fiber geometry on the properties of steel fiber reinforced concrete. Cem. Concr. Res..

[51] Glinicki M.A. Beton ze zbrojeniem strukturalnym. Proceedings of the XXV Ogólnopolskie Warsztaty Pracy Projektanta Konstrukcji.

[52] Balaguru P.N., Shah S.P. (1992). Fiber—Reinforced Cement Composites.

[53] Szmatuła J.K.F. (2023). Effect of Recycled Carbon Fiber Reinforcement on Concrete Properties. Proceedings of the Dni Betonu 2023.

[54] Overhage V., Gries T. (2025). Potential of and Current Challenges in Reusing Recycled Carbon Fibres in Concrete Construction Applications. Sustainability.

[55] Xiong C., Lan T., Li Q., Li H., Long W. (2020). Study of Mechanical Properties of an Eco-Friendly Concrete Containing Recycled Carbon Fiber Reinforced Polymer and Recycled Aggregate. Materials.

[56] de Souza Abreu F., Ribeiro C.C., da Silva Pinto J.D., Nsumbu T.M., Buono V.T.L. (2020). Influence of adding discontinuous and dispersed carbon fiber waste on concrete performance. J. Clean. Prod..

[57] Mastali M., Dalvand A., Sattarifard A. (2017). The impact resistance and mechanical properties of the reinforced self-compacting concrete incorporating recycled CFRP fiber with different lengths and dosages. Compos. Part B Eng..

[58] Lee J.-H. (2017). Influence of concrete strength combined with fiber content in the residual flexural strengths of fiber reinforced concrete. Compos. Struct..

[59] Wang C., Li K.-Z., Li H.-J., Jiao G.-S., Lu J., Hou D.-S. (2008). Effect of carbon fiber dispersion on the mechanical properties of carbon fiber-reinforced cement-based composites. Mater. Sci. Eng..

[60] Patchen A., Young S., Penumadu D. (2022). An Investigation of Mechanical Properties of Recycled Carbon Fiber Reinforced Ultra-High-Performance Concrete. Materials.

